# Minimum-Regret Hydrogen and Carbon Supply Chains to Decarbonize European Industrial Hydrogen Demands

**DOI:** 10.1021/acs.est.4c13659

**Published:** 2025-07-11

**Authors:** Alissa Ganter, Paolo Gabrielli, Hanne Goericke, Giovanni Sansavini

**Affiliations:** † 27219 Institute of Energy and Process Engineering, Zürich 8092, Switzerland; ‡ RWTH Aachen University, Aachen 52062, North Rhine-Westphalia, Germany

**Keywords:** hydrogen economy, uncertainty quantification, hard-to-abate industries, min-regret strategy, hydrogen supply chains, carbon dioxide capture, transport and storage, infrastructure

## Abstract

Low-carbon hydrogen (H_2_) is envisioned to play a central role in decarbonizing European hard-to-abate industries, such as refineries, ammonia, methanol, steel, and cement. To facilitate its widespread use, low-carbon H_2_ supply chain (HSC) infrastructure is needed. However, uncertainties around future low-carbon H_2_ demands and biomass availability hamper their proliferation. This work investigates the impact of uncertainties in H_2_ demand and biomass availability on the optimal HSC design. A linear optimization model is used to determine the cost-optimal HSC design, considering a regional spatial resolution and a multiyear time horizon from 2022 to 2050. CO_2_ and biomass infrastructure is designed alongside the HSC. A scenario-based approach is used to derive minimum-regret strategies and support infrastructure development. Results show that investing in scalable H_2_ production capacity, targeting 10 Mt/a by 2030, enables flexible expansion to meet larger H_2_ demands of up to 35 Mt/a by 2050. Although biomass-based H_2_ production is most cost-effective, coupling conventional H_2_ production with carbon capture and storage or investments in electrolysis provide more flexibility. Moreover, investments in CO_2_ infrastructure are essential, often determining the ability to achieve 2050 climate targets.

## Graphical abstract

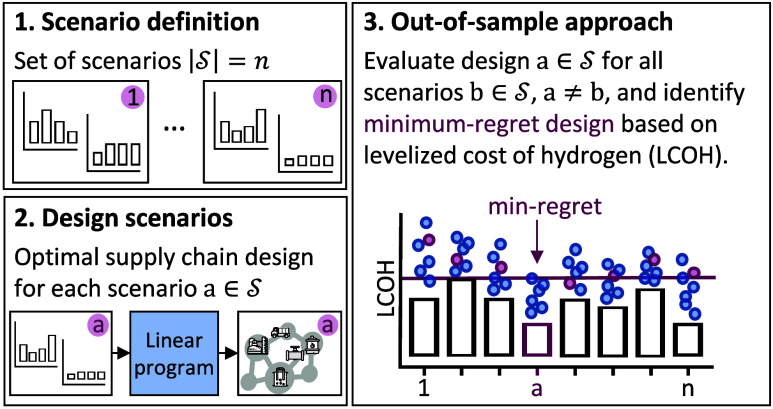

## 1 Introduction

The envisioned role of hydrogen (H_2_) in the future energy system has changed significantly throughout the years.^1,2^ Nevertheless, interest in H_2_ remains high, and its potential to decarbonize the industrial sector is widely acknowledged.^3,4^ The European industrial sector is currently responsible for 752 Mt (21%) of the annual anthropogenic greenhouse gas (GHG) emissions.^5^ Major contributors are the cement industry (15%), the iron and steel industry (14%), and the chemical industry, which includes refineries, methanol, and ammonia production (18%).^6^ These industries are difficult to decarbonize as they inherently rely on carbonaceous feedstocks and high-temperature heat, and therefore, are often referred to as “hard-to-abate” industries.

Efficiency improvements can reduce process emissions to an extent,^7,8^ however, additional measures are required to achieve the impending emissions targets, such as the EU Fit for 55 target, which requires a 55% emission reduction with respect to 1990, and the net-zero CO_2_ emissions target for 2050.^9^ Hence, a shift to low-carbon feedstocks and energy carriers is required. In this context, H_2_, produced with low CO_2_ emissions (i.e., low-carbon H_2_) is viewed as a promising solution. In Europe, H_2_ qualifies as “low-carbon” if process emissions are below 3.38 kg_CO_2_eq_/kg_H_2__. This corresponds to a 70% reduction in GHG emissions compared to fossil-fuel-based H_2_ production processes which emit about 11.3 kg_CO_2_eq_/kg_H_2__.^10^

About 9% (8.2 Mt) of the global H_2_ demand is currently (2022) produced and consumed in Europe,^11^ 85% of which is used as a feedstock to produce ammonia, methanol, and other chemicals, or in refineries.^12^ Aside from the current use of H_2_ as a feedstock, low-carbon H_2_ has the potential to replace coal as a reducing agent in the steel-making process,^13^ to generate high-temperature heat required in the cement-making process,^14^ or, combined with captured CO_2_, to replace carbonaceous feedstocks in chemicals production (e.g., methanol and plastics).^15^

However, the future industrial low-carbon H_2_ demand is deeply uncertain and current literature presents a divided outlook. While some studies suggest industrial H_2_ demands to grow, reaching up to 40 Mt/a by 2050,^13,16^ others anticipate demands to fall below 3 Mt/a by 2050.^17^ While the EU envisions a large-scale H_2_ infrastructure to enable its widespread use,^18,19^ the large uncertainty around the low-carbon H_2_ demands obstructs planning^20–22^ and hinders investments.^23^ Developing a minimum-regret strategy can help mitigate risks and identify investment opportunities that retain their value across demand scenarios.

In general, low-carbon H_2_ can be produced starting from renewable electricity via water-electrolysis and from biomass via biomass gasification or biomethane reforming.^24^ Furthermore, H_2_ production from natural gas via steam methane reforming coupled with CO_2_ capture and storage (CCS) can be considered low-carbon if CO_2_ capture rates exceed 90% and leakage rates of natural gas supply chains below 0.2%.^25^ A comparison of the available low-carbon H_2_ production routes identifies biomass-based H_2_ production as the most cost-effective alternative while offering large reductions in CO_2_ emissions; even enabling CO_2_ removal when coupled with CCS.^26^ However, difficulties in biomass collection, a lack of infrastructure, and competing interests with other sectors may substantially reduce the amount of biomass that can be dedicated for low-carbon H_2_ production.^27–29^ While recent evidence suggests that it is less important where biomass is used, as long as it is combined with CCS to enable CO_2_ removal,^30^ it remains uncertain which sectors will gain access to the limited biomass resources. This additional uncertainty can hinder investments and further delay the infrastructure rollout. Therefore, the uncertainty in biomass availability should be considered when planning low-carbon HSCs.

Energy system optimization models have proven to provide useful insights for energy system planners and policymakers.^31^ They are widely used to investigate HSCs, offering an integrated representation of H_2_ production and transport, and enabling the analysis of trade-offs between technology alternatives over long-term, multiperiod time horizons.^32^ However, a key challenge lies in the uncertainty associated with the input data of energy system optimization models,^33^ and accuracy assessments reveal that energy system optimization models systematically underestimate uncertainties.^34–36^ Therefore, the uncertainty should be accounted for in the decision-making process to increase robustness and derive long-term policy recommendations.^33,37^

HSC models are already object of extensive literature reviews.^38,39^ While most HSC models are deterministic, several works exist that include uncertainty, with refs 40,41 leading the way. Thus, far, many studies pertain to the uncertainty in the H_2_ demand, e.g., refs 21,42–44 which is identified as the most influential parameter for the HSC design.^20^

Four approaches are commonly used to address the uncertainty in the input data: (1) Monte Carlo analysis (e.g., ref 43), (2) stochastic programming (e.g., refs 41,45 ), (3) robust optimization (e.g., ref 46), and (4) scenario-based uncertainty analysis (e.g., ref 47). While (1)-(3) provide clear recommendations to decision-makers by directly accounting for the uncertainty in the input data, they also largely increase the model complexity making it difficult to maintain feasibility. Considering computational limitations, scenario-based approaches are often better suited to include uncertainty in large-scale energy system models.^33^ Min-max regret criteria can be used to hedge against parameter variations and identify the solution that performs best, even in the worst case.^48^

To the best of our knowledge, no uncertainty analysis of the European HSC infrastructure rollout exists to date. This work aims to address this gap and identifies minimum-regret strategies that result in the lowest costs considering the deep uncertainty surrounding the future H_2_ demand and the availability of biomass feedstocks for H_2_ production. In particular, we investigate (1) how uncertainties in the future H_2_ demand and biomass feedstock availability influence the optimal design and rollout of HSC and CO_2_ infrastructures, and (2) what H_2_ production technologies, feedstocks, and energy sources are consistently deployed in the optimum HSC infrastructure design of the future.

The paper is structured as follows. Section 2 describes the considered system (Section 2.1), the uncertainty quantification approach for H_2_ demand and biomass availability (Sections 2.2 and 2.3, respectively), and the solution strategy used to investigate the optimal HSC under uncertainty and identify the minimum-regret strategy (Sections 2.4 and 2.5, respectively). Section 3 presents the results and Section 4 discusses their implications. Finally, Section 4.5 draws conclusions.

## 2 Optimal Design of Hydrogen Supply Chains (HSCs) under Uncertainty

The elements of the analyzed HSCs are described in Section 2.1. Furthermore, Figure 1 provides an overview of the methodology developed to identify the minimum-regret HSC design considering uncertainties in future industrial H_2_ demands and biomass availability. A scenario-based approach is used to maintain the computational tractability of the large-scale HSC model. “Scenario definition” derives a discrete set of scenarios S by combining the H_2_ demand and biomass availability projections (Sections 2.2 and 2.3). “Design scenario” determines the optimal HSC design for each scenario a∈S using a deterministic, linear optimization model outlined in Section 2.4. The resulting HSC design describes the least-cost low-carbon H_2_, CO_2_, and biomass infrastructure rollout for a given H_2_ demand and biomass availability. “Out-of-sample approach” evaluates the HSC designs derived in “Design scenario”, when operated under all other possible scenarios b∈S,b≠a .

**Figure 1:**
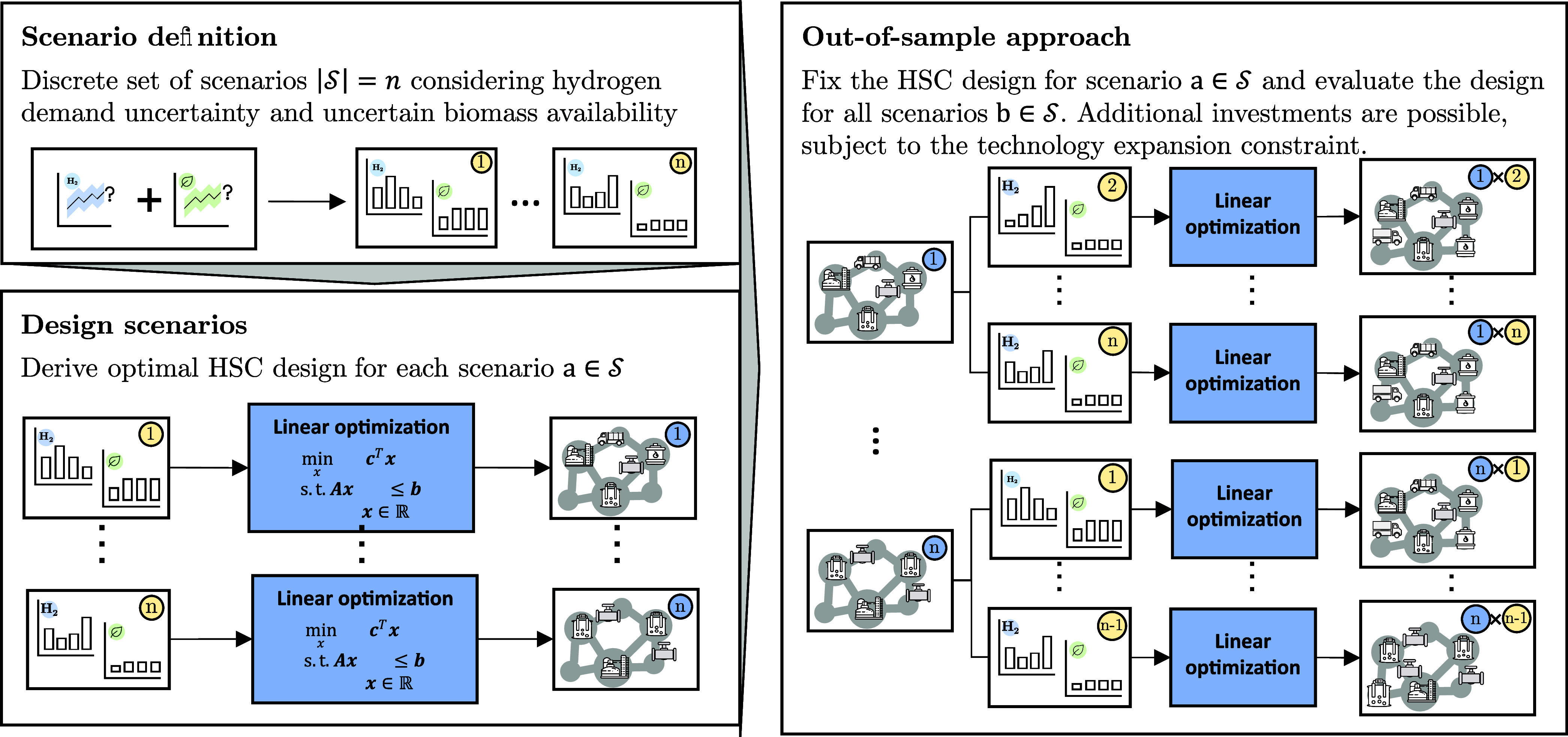
Solution strategy adopted to identify the minimum-regret H_2_ supply chain design considering uncertainties in the evolution of the future industrial H_2_ demand and the availability of biomass.

Additional investments may be needed to adapt the initial supply chain design derived under scenario *a* to meet the target decarbonization pathway under the new conditions of scenario *b*. However, these additional investments, and thus, the speed at which technology capacities can be expanded, are limited by the existing and planned capacities of the technology manufacturers.^49^ We include this by adding a technology expansion constraint to the optimization model, which limits the speed at which technology capacity can be expanded based on the existing capacity stocks (Sections 2.4 and S3) and test, whether it is possible to adapt a given HSC design to meet the decarbonization target under the new conditions.

Finally, the performance of each design scenario of the HSC is evaluated based on the levelized cost of H_2_ (LCOH) of the out-of-sample scenarios and the minimum-regret solution is identified. The goal is to select the supply chain design that exposes decision-makers to the least negative consequences. Therefore, only designs are considered that can be adapted to fulfill the annual emission targets and for which costs remain the lowest (min-max LCOH, Section 2.5).

### 2.1 System Description

The HSC is modeled as a network of nodes and edges, with regional resolution following the EU’s Nomenclature of territorial units for statistics for level 2 (NUTS2).^50^ At each node, H_2_ can be produced through a portfolio of feedstocks and energy sources, including natural gas, electricity, and biomass. To reduce CO_2_ emissions, H_2_ production from natural gas and biomass can be coupled with CO_2_ capture technologies. Edges connect the nodes. The distance between two nodes is approximated by their Haversine distance. At each edge, H_2_, CO_2_, and biomass transport technologies can be installed. In the following text, the individual components of the supply chain are described in more detail. Figure 2 provides an overview of the available feedstocks and energy sources, the available H_2_ production and transport technologies, and the considered industrial H_2_ demands. The full model description is published in ref 51. Moreover, the input data is detailed in Section S1.

**Figure 2:**
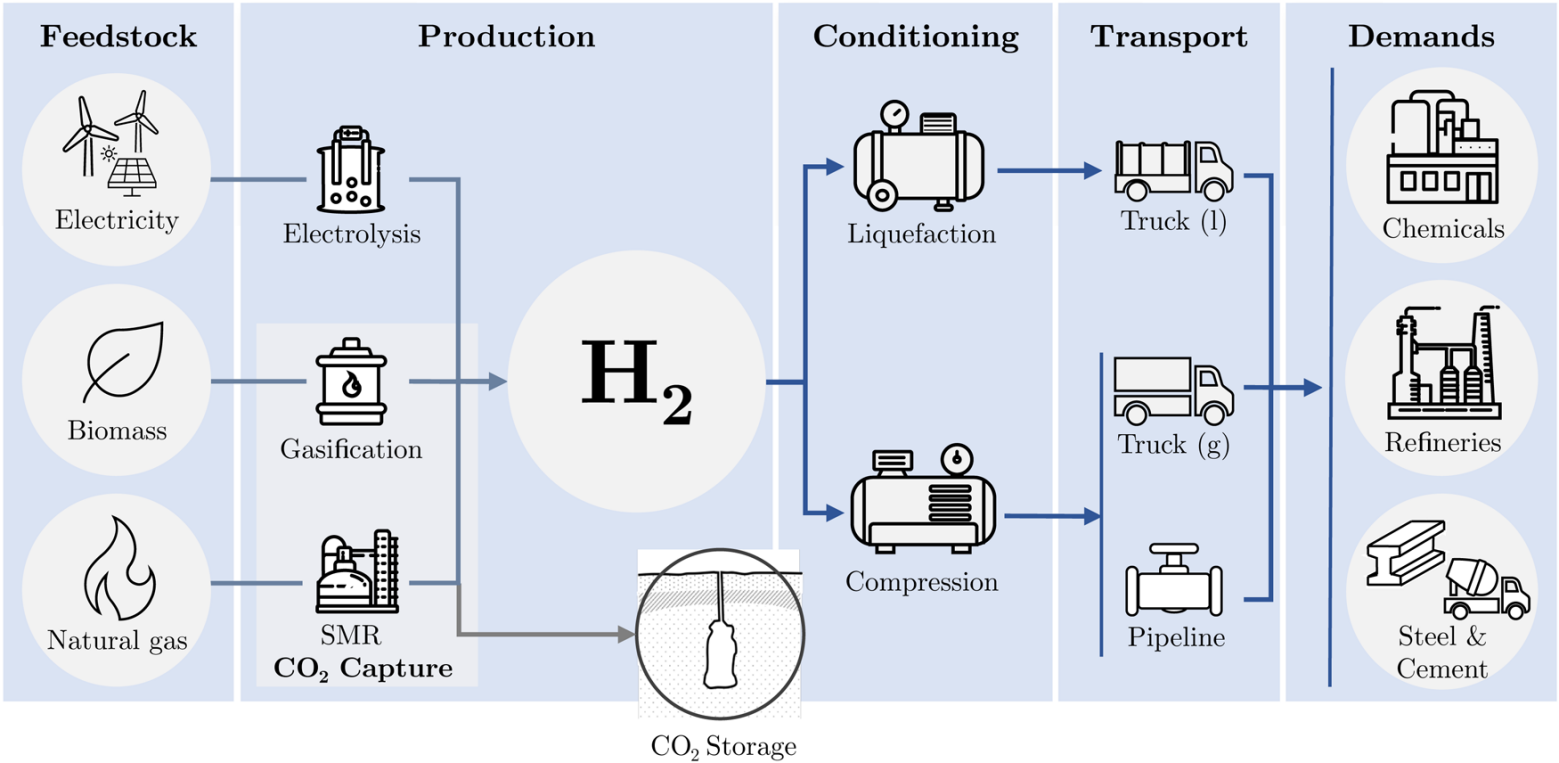
Overview of the available feedstocks and energy sources, the available H_2_ production, conditioning, and transport technologies, and the considered industrial H_2_ demands.

#### 2.1.1 Feedstocks and Energy Sources

The considered feedstocks and energy sources are natural gas, electricity, and biomass. We assume that natural gas and grid electricity are available at each node. Furthermore, renewable electricity can be generated from wind and solar energy. The wind and solar energy potentials are modeled as the technical potentials reported in refs 52,53 . Furthermore, the availability of biomass is subject to uncertainty and limited according to the selected scenario (Section 2.3).

#### 2.1.2 Hydrogen Production Technologies

The available H_2_ production technologies are (i) steam methane reforming (SMR) from natural gas or biomethane, (ii) water-electrolysis from electricity, and (iii) biomass gasification. SMR and biomass gasification can be coupled with CO_2_ capture and storage to lower process emissions. In addition, direct-air-capture capacities can be installed to generate carbon dioxide removal and offset emissions that occur during H_2_ production or transport. The techno-economic parameters of the H_2_ production technologies are reported in Section S1.2.

#### 2.1.3 Hydrogen Transport Technologies

The available H_2_ transport technologies are trucks and pipelines. The transport conditions for H_2_ vary depending on the transport mode. Trucks transport H_2_ in ISO-tank containers (isotainers) as a compressed gas or in its liquid form (H_2_ truck gas and H_2_ truck liquid), and pipelines transport H_2_ as a compressed gas. To meet the specific transport requirements, H_2_ is conditioned (i.e., compressed or liquefied). The techno-economic parameters of the H_2_ transport and conditioning technologies are reported in Section S1.2.

#### 2.1.4 Hydrogen Demand

Current (2020) and potential future H_2_ demands for ammonia production, methanol production, refineries, steel production, and cement production are considered. To account for the uncertainty in future industrial H_2_ demands, different H_2_ demand scenarios are investigated (Sections 2.2 and S2).

#### 2.1.5 CO_2_ Supply Chain

A CO_2_ supply chain is designed alongside the H_2_ supply chain to account for the transport of the captured CO_2_ to the offshore storage locations. We consider 27 potential CO_2_ storage locations in Europe following ref 54. The CO_2_ storage capacity is limited by the capacity of existing and announced CO_2_ storage projects, which corresponds to 132 Mt/a.^54^ The available CO_2_ transport technologies are trucks (with isotainers) and pipelines. Similarly to H_2_, the captured CO_2_ is conditioned to meet transport requirements. The techno-economic parameters of the CO_2_ transport technologies are reported in Section S1.2.

#### 2.1.6 Biomass Supply Chain

Two types of biomass are considered: dry and wet biomass. Dry biomass consists of woody biomass, which can be transported for long distances via containers loaded on trucks.^28,55^ Dry biomass serves as a feedstock for biomass gasification to produce hydrogen. Wet biomass consists of manure and waste biomass, whose collection and transport is challenging,^28^ with maximum transport distances ranging between 10 to 50 km.^28,56^ Therefore, we assume that wet biomass cannot be transported and is only used at the locations where it is available. Wet biomass serves as feedstock for anaerobic digestion to produce biomethane, which can be transported via isotainers loaded on trucks^57^ or injected into the natural gas grid.^13^ Further detail on the assumptions around biomass is provided in ref 51. Moreover, we assume that the gas grid is available at each node of the supply chain, and that biomethane can be injected into the gas grid subject to grid connection costs.^13^ The techno-economic parameters for dry biomass transport and biomethane transport are reported in Section S1.2.

### 2.2 Hydrogen Demand Uncertainty

The uncertainty analysis covers H_2_ demand predictions for European hard-to-abate industries, namely refineries, ammonia, methanol, steel, and cement industries. A literature search is conducted to collect H_2_ demand forecasts for the different industries and covers publications from 2018 to today. In total, 28 literature scenarios are analyzed (Table 1). Scenarios that do not provide a breakdown of the H_2_ demand estimates for the considered industries are excluded from further analysis.

**Table 1:** Hydrogen Demand Projections in Literature for Hard-to-abate Industries in 2050

**title**	**no. scenarios**	**2050 demand [Mt/a]**	**industries**	**source**
12 insights on hydrogen	2	6.6–14.5	chemicals, steel, refineries	^62^
no-regret hydrogen	1	8.1	chemicals, steel, refineries	^17^
navigating through hydrogen	3	8.0–18.6	chemicals, steel, refineries	^63^
clean planet for all	11	0–18.5	chemicals, steel, refineries, cement	^64^
hydrogen roadmap	2	2.5–5.6	steel	^65^
the optimal role for gas in a net zero emissions energy system	1	9.9	chemicals, steel, refineries, cement	^13^
analyzing future demand, supply, and transport of hydrogen	1	22	chemicals, steel, refineries, cement	^14^
the potential of hydrogen for decarbonising EU industry	2	6.3–18.8	chemicals, refineries, steel	^66^
hydrogen roadmap europe	2	12.2–19.6	chemicals, steel, refineries, cement	^67^
industrial transformation 2050	3	5.6–10.7	chemicals, steel, cement	^58^

The feasibility and attractiveness of hydrogen-based solutions is influenced by technological, economic, and political factors. Uncertainties surrounding technological factors such as technological breakthroughs and the time required for commercialization, as well as uncertainty surrounding economic factors such as the cost-competitiveness of low-carbon H_2_ technologies and the uncertainty surrounding future cost trajectories can hinder their adoption. In contrast, political measures such as CO_2_ pricing mechanisms can address affordability issues and promote investments.^23,32^

In particular, assumptions surrounding (1) material and process efficiency, (2) electrification, (3) recycling, and (4) CO_2_ capture and storage strongly influence the H_2_ demand predictions.^58^ For example, in ammonia production, material and process efficiency improvements could reduce H_2_ feedstock requirements by up to 25% with respect to today’s values.^58^ In steel industry, recycled (or secondary) steel could replace 50–70% of the primary steel production, thereby reducing potential future industrial H_2_ demands substantially.^59^ The availability of electricity-based decarbonization pathways and the option of CO_2_ capture and storage add further layers of uncertainty.^60^

Figure 3a shows the 2050 H_2_ demand estimates for the considered industries reported across literature (Table 1). In general, the spread of projected industrial H_2_ demands is lower in industries that inherently rely on H_2_ as a feedstock in their production process (ammonia production plants and refineries). The spread increases for industries where multiple decarbonization strategies compete with each other (e.g., steel and cement industry). The largest spread is observed for methanol, an important base chemical that is required e.g., for plastics production via the methanol-to-olefins route. While refs 13,14 expect a large uptake of methanol-to-olefins, and thus, the methanol demand,^17^ provide more conservative methanol demand estimates. Section S2 provides a detailed analysis of the variability in the regional H_2_ demand estimates.

**Figure 3:**
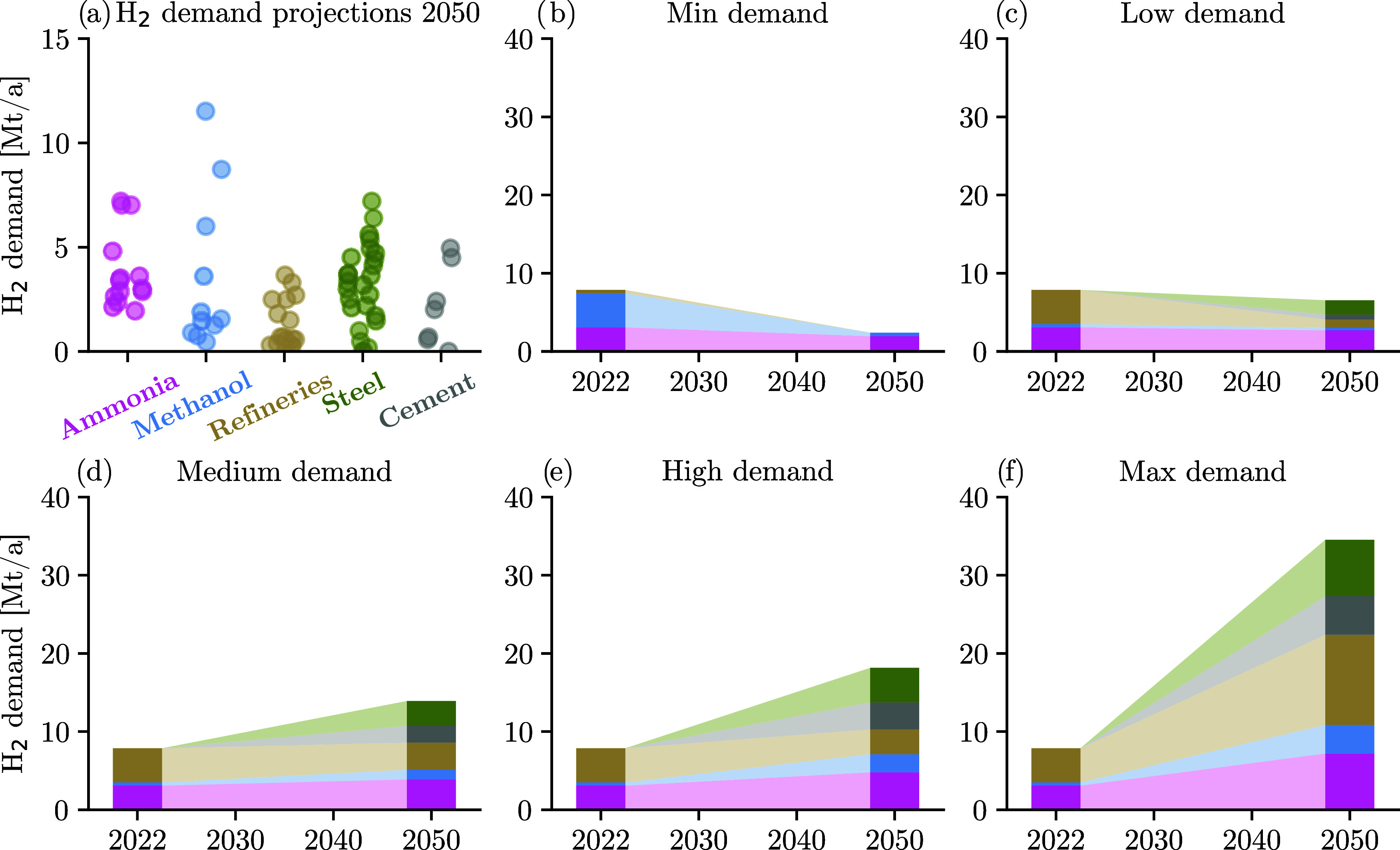
(a) European H_2_ demand estimates reported in the literature scenarios for refineries, ammonia, methanol, steel, and cement industry in 2050. (b–f) European H_2_ demand per industry in the minimum, low, medium, high, and maximum H_2_ demand scenario.

Five H_2_ demand scenarios are developed to tackle the deep uncertainty associated with the future industrial H_2_ demands: minimum (min), low, medium (med), high, and maximum (max). The medium demand scenario represents the average of the H_2_ demand estimates. The low and high demand scenarios are derived based on the 25th and 75th quartile of the H_2_ demand estimates. Quartiles are used here because they are insensitive to outliers, but maintain the information about the center and spread of the H_2_ demand estimates.^61^ Finally, the min and max demand scenarios represent the minimum and maximum H_2_ demand estimates and are added to cover the full range of the H_2_ demand estimates. Figure 3b–f visualize the temporal evolution of the five H_2_ demand scenarios, where data is collected for 2020 and 2050, and linear interpolation is used for intermediate years.

### 2.3 Biomass Availability Uncertainty

Refs 55 and 68 estimate the technical and sustainable potential, respectively, of bioenergy for different types of biomass in Europe. However, the availability of biomass feedstocks is highly uncertain, and sustainability and socio-political factors such as the competition of biomass feedstocks with food production or alternative land uses are often not accounted for.^69^ Here, we focus on sustainable biomass potentials, i.e., biomass that is not primarily grown for energy use and does not compete with food production or alternative land uses. Sustainable biomass includes residues from agriculture and forests and animal manure.^70^

To account for the uncertainty in biomass availability, we define three discrete scenarios: a reference scenario, a scenario with reduced biomass availability to account for competition with other sectors, and a scenario where no biomass is available for low-carbon H_2_ production. In the reference scenario, the estimates from ref 68 are used. In the scenario with reduced biomass availability, we assume that the sectoral biomass consumption is proportional to the sectoral primary energy demands. According to the EU reference scenario, 26% of the total energy consumption can be attributed to industry. Thus, in the reduced biomass scenario, only 26% of the sustainable biomass potential is available for industrial H_2_ production.

### 2.4 Optimization Model

The least-cost HSC design is determined via a linear optimization problem following ref 51. In its general form, a linear optimization problem can be formulated as follows:

1 $$\begin{array}{ll} \min_{x} & c^{T}x\\ s.t. & \mathrm{Ax}\leq b\\ & x\in R^{N} \end{array}$$

where the objective function is expressed as a linear combination of continuous decision variables **x** with dimension *N* and coefficients **c**; and the constraints are expressed as a linear combination of matrix **A**, decision variables **x**, and vector **b**.

The optimization problem is implemented in the optimization framework ZEN-garden (Zero-emissions Energy Networks), developed at the Reliability and Risk Engineering Lab at ETH Zurich. ZEN-garden optimizes the design and operation of energy system models to investigate transition pathways toward decarbonization.^51,71,72^ The optimization problem is solved using the commercial solver Gurobi.^73^

#### 2.4.1 Input Data

The input data to the optimization problem includes (i) spatially resolved industrial H_2_ demands, carrier prices, CO_2_ intensities, and availabilities of biomass, wind, and solar energy, (ii) the techno-economic parameters describing the cost and performance of production, conditioning, and transport technologies, (iii) the existing H_2_ production capacities, (iv) the size and location of the available CO_2_ storage sites, and (v) the target decarbonization pathway. A yearly resolution is used to model the time-dependent variables, namely the industrial H_2_ demands, carrier prices, CO_2_ intensity of the electricity grid, and biomass availability. Moreover, the input data is resolved at a regional level, following the EU’s NUTS2 classification.^50^ The input data is reported in Section S1.

#### 2.4.2 Decision Variables

The optimization problem determines (i) the optimal selection, capacity, and location of the H_2_ production, conditioning, and transport technologies, (ii) the energy inputs and outputs of each H_2_ production and conditioning technology, (iii) the carrier flows through each transport technology, (iv) the nodal carrier imports and exports.

#### 2.4.3 Constraints

The constraints of the optimization problem include (i) the nodal mass balances for electricity, natural gas, H_2_, biomass, and CO_2_, (ii) the performance and operating limits of the H_2_ production, conditioning, and transport technologies, and (iii) the CO_2_ emissions constraint limiting the yearly CO_2_ emissions to the target emissions values of the selected decarbonization pathway. Here, we assume linearly decreasing CO_2_ emissions from today’s values such that the 2050 net-zero emissions (NZE) target is achieved.^74^ To ensure the feasibility of the optimization problem, a slack variable is added to the CO_2_ emission constraint. This slack variable can be interpreted as a CO_2_ emissions overshoot of the emission target and is associated with a large cost (100k€/t), ensuring that overshooting the CO_2_ emission constraint is selected as a last resort. The HSC design is considered a feasible solution if the CO_2_ emissions target is achieved and the CO_2_ emissions overshoot is zero.

The constraints are detailed in ref 51. Here, we expand the model formulation from ref 51 by adding technology expansion constraints when performing the out-of-sample approach. The technology expansion constraints limit the maximum annual growth rate of a technology based on the existing capacity stock and are formulated following refs 49,72 and described in Section S3. The technology expansion is parametrized based on historically observed growth rates.^72^ investigate the historical annual growth rates for low-carbon technologies and observe growth rates between 10% (wind offshore) to 29% (solar PV). For our reference case, we select a technology expansion rate of 20%. A sensitivity analysis is performed for technology expansion rates varying from 10 to 29%.

#### 2.4.4 Objective Function

The optimization problem minimizes the net present cost of the system, which includes the investment and operating costs of the H_2_, CO_2_, and biomass supply chains in compliance with the CO_2_ emission targets. A discount rate of 6% is used.

### 2.5 Minimum-Regret Solution

The minimum regret solution is the HSC scenario design that (i) results in the lowest levelized cost of H_2_ (LCOH) for the worst-case out-of-sample scenario (i.e., the min-max across the LCOH of the out-of-sample scenarios), and (ii) is feasible for all scenarios. The LCOH is computed as the net present costs of the system divided by the net present H_2_ production from 2022 to 2050. The net present costs include the H_2_, biomass, and CO_2_ supply chain costs. Finally, feasibility is defined as the ability to meet the annual CO_2_ emissions targets, i.e., the CO_2_ emission overshoot is zero.

## 3 Results

The combination of five H_2_ demand levels and three biomass availabilities produces 15 scenarios. Section 3.1 analyzes the optimal investment strategies across scenarios. Section 3.1 contrasts the H_2_ and CO_2_ transport infrastructure requirements, and Section 3.3 compares the levelized cost of H_2_ (LCOH) across the 15 design scenarios and evaluates their ability to fulfill the decarbonization pathway if one of the 14 other scenarios materializes, using the out-of-sample approach. Finally, Section 3.4 discusses the characteristics of the minimum-regret solution.

### 3.1 Optimal Investment Strategies for Different Levels of Hydrogen Demand and Biomass Availability

Figure 4 shows the cost-optimal rollout of the H_2_ production, and CO_2_ capture and storage capacities across all scenarios. The installed H_2_ production capacity depends on the expected H_2_ demand. In the max H_2_ demand scenario, the H_2_ production capacity for 2050 is about 7 times higher than in the min H_2_ demand scenario. In contrast, the H_2_ production technology mix and the CO_2_ capture and storage capacities strongly depend on the availability of biomass.

**Figure 4:**
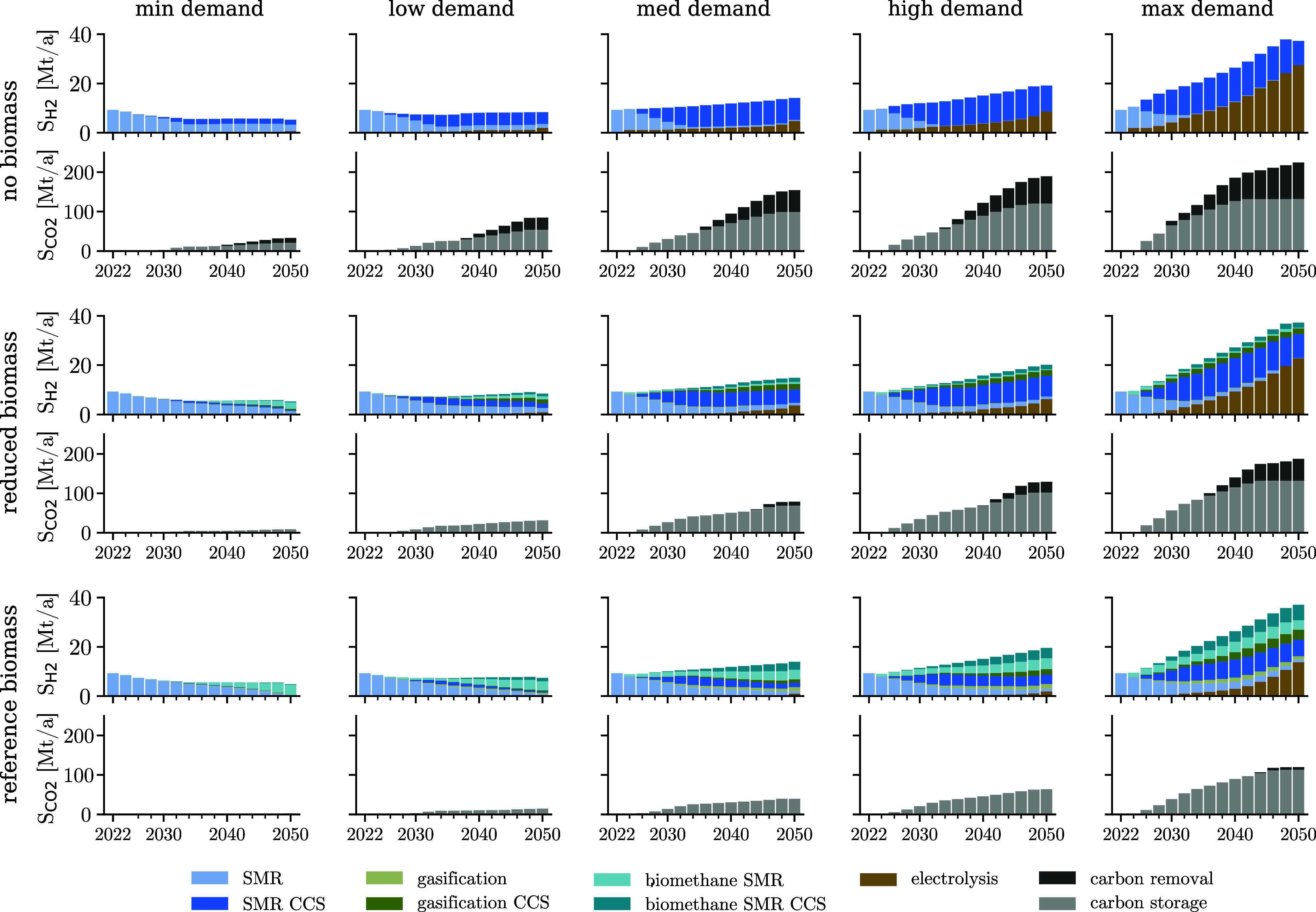
Cost-optimal rollout of H_2_ production and CO_2_ capture and storage capacity (*S*_H_2__ and *S*_CO_2__) in Mt/a from 2022 to 2050 across the 15 design scenarios. H_2_ production technologies include steam methane reforming (SMR) from natural gas, biomethane reforming, biomass gasification, and water-electrolysis from electricity. SMR, biomethane reforming, and biomass gasification can be coupled with CCS. In addition, CO_2_ removal and CO_2_ storage capacity can be installed to meet the net-zero emissions target by 2050. Existing SMR capacity is coupled with CCS to reduce emissions. Moreover, CO_2_ removal and CO_2_ storage capacity is deployed across all scenarios to achieve the 2050 net-zero emission target.

Biomass-based H_2_ production is identified as the most cost-efficient low-carbon H_2_ production pathway. By replacing natural gas with biomethane, the SMR process emissions can be reduced by 82%. Besides installing anaerobic digesters to produce biomethane, no additional investments are required in the system considered, and existing SMR capacities can continue to be used. Furthermore, the coupling of biomass-based H_2_ production with CCS results in net-negative emissions, offsetting CO_2_ emissions that occur at other stages of the supply chain. Therefore, as a first step, scenarios with biomass availability replace natural gas feedstocks with biomethane. With increasing annual decarbonization targets, H_2_ production is complemented with CCS to reduce process emissions and biomass gasification is deployed. For max H_2_ demands, the reference biomass potentials are insufficient to fully decarbonize H_2_ production, and increasing shares of electrolyzers are installed. In addition, we observe investments in CO_2_ removal technologies to offset upstream supply chain emissions from fuel supply chain, plant manufacturing and construction phases (about 6 Mt/a). The same considerations apply for scenarios with reduced biomass availability and medium-max H_2_ demands, which require between 10 and 56 Mt/a CO_2_ removal capacities by 2050 to achieve the net-zero emissions target.

Without biomass feedstock, low-carbon H_2_ is produced via SMR-CCS from natural gas and electrolysis of renewable electricity. In addition, CO_2_ removal technologies are installed to eliminate residual emissions and achieve the NZE target. In configurations where the H_2_ demand is expected to decrease or remain similar to today’s values (i.e., min-low demand scenarios, Figure 4), H_2_ is predominantly produced via SMR-CCS, which is associated with larger CO_2_ emissions, but lower cost compared to electrolysis. Electrolyzer capacities are continuously expanded in scenarios where H_2_ demand is expected to increase.

Independently of the biomass availability, electrolyzers do not play a role in scenarios with min-low H_2_ demand and are only deployed if medium-max demands are expected. Two factors contribute to the increasing deployment of electrolyzers. First, the unit cost of electrolyzers is expected to decrease by 60% until 2050, making electrolyzers more cost-competitive. Second, the residual emissions of electrolyzers are between 4–10 times lower compared to SMR-CCS (0.6–1.6 ton_CO_2_eq_/ton_H_2__ vs 5.9 ton_CO_2_eq_/ton_H_2__). Hence, offsetting the residual emissions from electrolysis requires significantly smaller capacities of CO_2_ removal technologies and CO_2_ storage. In literature, a wide range of electrolyzer cost estimates is reported. However, even when assuming a very optimiztic cost evolution for electrolyzers, where the investment costs are 10% lower in 2022 and 30% lower in 2050 with respect to the reference case, electrolyzer capacities remain small and are only deployed at a larger scale in high and max H_2_ demand scenarios (Section S7).

The CO_2_ removal technologies are located near the CO_2_ storage sites to reduce CO_2_ transport costs. Land requirements for CO_2_ removal technologies range between 0.2 and 0.4 km^2^.^75,76^ Depending on the scenario the capacity of the CO_2_ removal technologies range between 6 to 92 Mt/a which translates to 1.2–18 km^2^ in the best case, and 2.4–37 km^2^ in the worst case. By far, the largest CO_2_ removal capacity is installed close to the Northern Lights CO_2_ storage site in Norway where about 30 Mt/a CO_2_ are removed from the air each year, requiring an area of 6–12 km^2^ (about 840–1680 soccer fields). To provide more context, this corresponds to less than 1% of the open free available area.^77^ In the remaining regions, the CO_2_ removal capacity is below 8.5 Mt/a (1.7–3.4 km^2^).

Finally, the regional CO_2_ storage capacity is limited by the capacity of existing and announced CO_2_ storage projects, which corresponds to 132 Mt/a.^54^ The full CO_2_ storage potential is used by 2050 if max H_2_ demands are expected and less than the reference biomass potentials are available for H_2_ production.

### 3.2 Hydrogen and Carbon Dioxide Transport Networks

A H_2_ transport network is built to decouple H_2_ production and demand. Moreover, a CO_2_ transport network is added to transport the captured CO_2_ to the CO_2_ storage sites. Figure 5 compares the H_2_ and CO_2_ transport networks in 2050 with respect to (i) the network capacity (*x* and *y* location of the bubble for H_2_ and CO_2_, respectively), and (ii) the network extension (size of the right and left half of the bubble for H_2_ and CO_2_, respectively). The capacity of the H_2_ and CO_2_ networks is strongly connected to the H_2_ demands, and in general, the transport volumes and the network capacities increase with increasing H_2_ demands.

**Figure 5:**
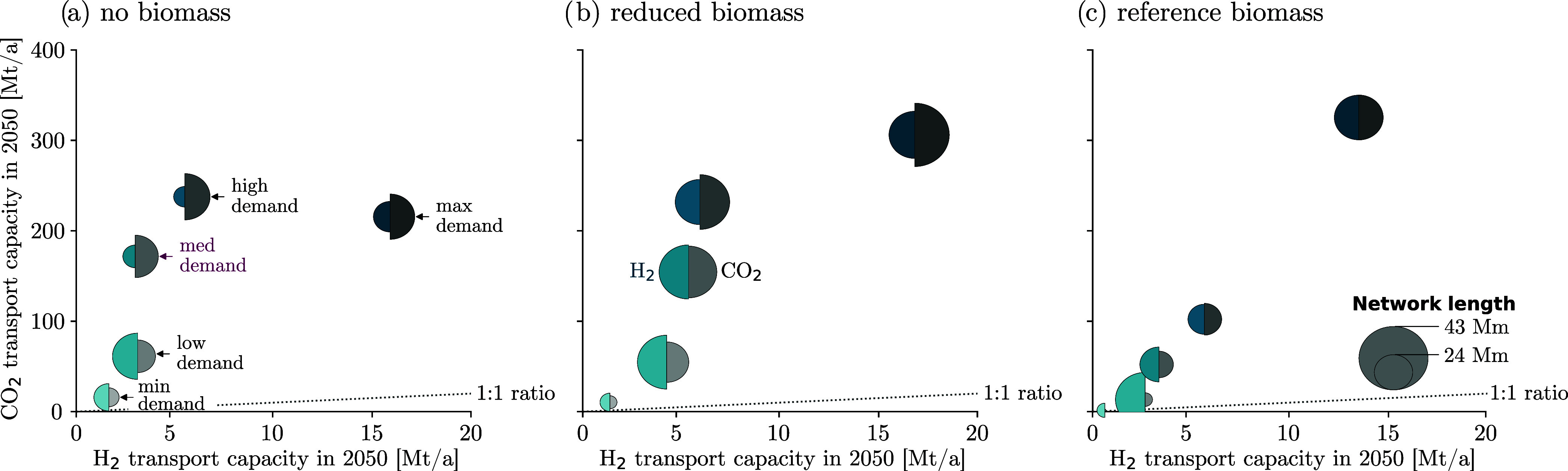
H_2_ and CO_2_ transport network capacity and length for the 15 design scenarios. The location of the bubbles indicates the cumulative H_2_ (*x*) and CO_2_ transport network capacity (*y*). The bubble size indicates the H_2_ (left, petrol color) and CO_2_ network length (right, gray color). The shading indicates the level of H_2_ demand, where lighter shades indicate lower demands and darker shades indicate higher demands. We observe that the choice of H_2_ production method ((a) no biomass vs (b) reduced and (c) reference biomass) significantly impacts the scale of the H_2_ and CO_2_ transport infrastructure.

While the H_2_ network transport capacity is comparable for different levels of biomass, the spatial extension of the network varies greatly. System designs that rely on biomass deploy larger H_2_ transport infrastructure to overcome the additional spatial constraints imposed by the heterogeneous biomass availabilities and, therefore, install more widespread transport infrastructures. To enable CCS, CO_2_ transport infrastructure is installed alongside the HSC to transport the captured CO_2_ to the storage sites. While the capacity of the CO_2_ networks for scenarios with reference biomass and no biomass is comparable, larger distances are covered in scenarios with reduced biomass potentials to access the limited biomass resources and enable the coupling with CCS.

In contrast, system designs that do not rely on biomass produce H_2_ closer to the demand locations, resulting in smaller, more local H_2_ transport networks. In these cases, H_2_ is largely produced via SMR-CCS from natural gas, and electrolysis from renewable electricity, and CO_2_ removal technologies are installed to offset residual emissions from H_2_ production and upstream emissions from plant manufacturing and construction (Figure 4). In earlier years and for lower demands, H_2_ is predominantly produced via SMR-CCS. However, for larger H_2_ demands, SMR-CCS is complemented by increasing shares of electrolyzers, reducing the need for CO_2_ transport from the H_2_ production site to the CO_2_ storage locations (compare infrastructure size for min-low demand and medium-max demand for no biomass in Figure 5a).

### 3.3 Levelized Cost of Hydrogen for Different Levels of Hydrogen Demand and Biomass Availability

Figure 6 presents the LCOH (i) for each design scenario (i.e., the system is designed and operated on the same scenario; see stacked bars) and (ii) the results of the out-of-sample approach (i.e., the system is designed for one scenario and operated on all other 14 scenarios; see markers above each bar). The marker shape and color indicate which out-of-sample scenario the supply chain design is operated on. Furthermore, a red marker edge indicates that the out-of-sample scenario is infeasible, i.e., it is impossible to adjust the system design quickly enough such that the annual CO_2_ emissions targets are always satisfied.

**Figure 6:**
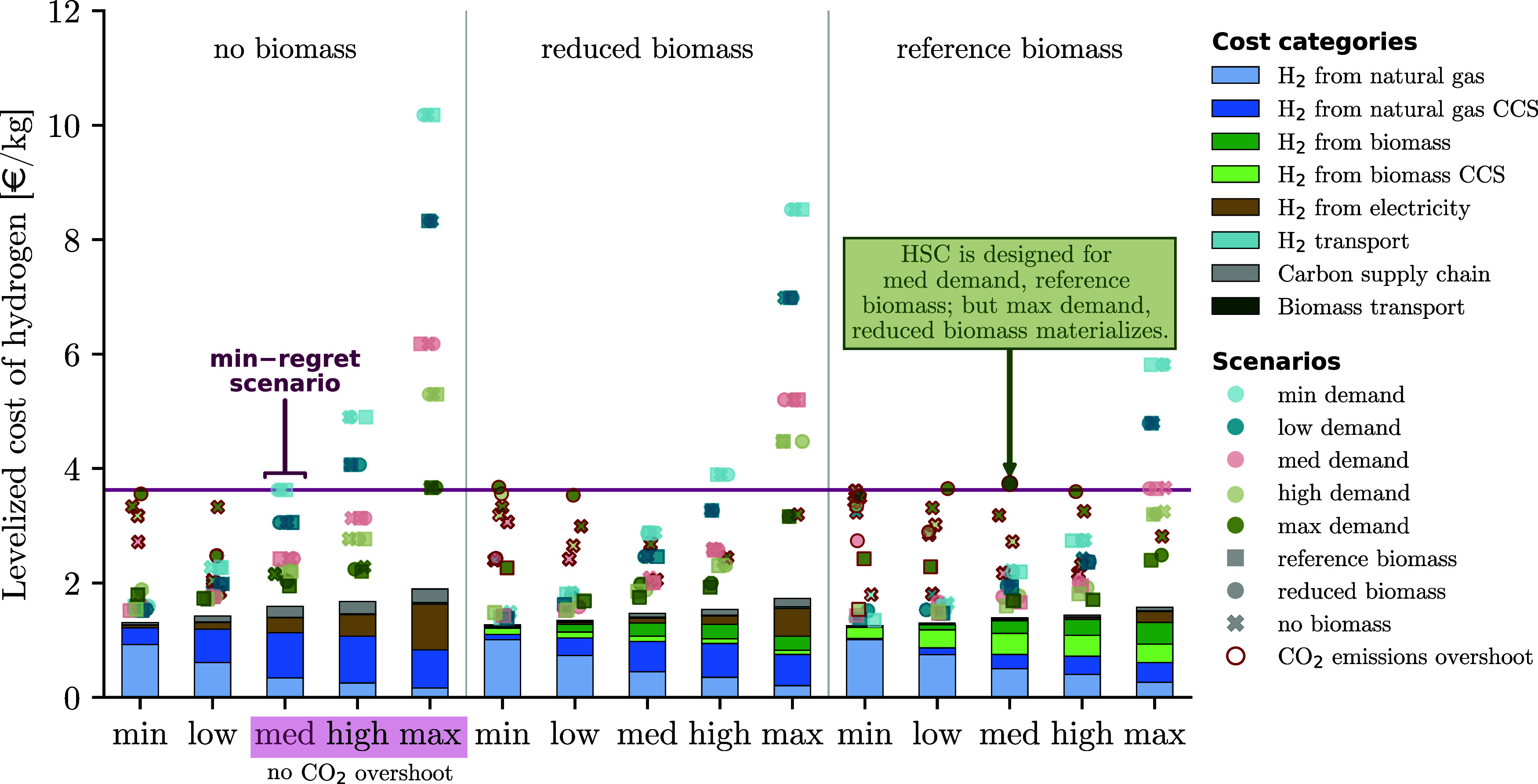
Levelized cost of H_2_ (LCOH) across all scenarios s∈S . The LCOH is computed as the net present cost divided by the net present H_2_ production. The bars show the LCOH of the design scenarios. The markers above the bars show the LCOH that arises if the supply chain is initially designed for scenario a∈S , but scenario b∈S materializes. A red marker edge color indicates infeasible scenarios where the annual CO_2_ emissions targets cannot always be fulfilled. The minimum-regret solution is identified based on two criteria: (1) the solution results in the lowest LCOH in the worst scenario (min-max cost criteria), while (2) meeting the annual CO_2_ emissions targets (feasibility criteria). The horizontal line indicates the highest LCOH of the minimum-regret scenarios. Although utilizing biomass for H_2_ production initially results in lower costs, it is difficult to adapt the infrastructure if different conditions materialize. Therefore, planning without biomass is recommended, despite higher upfront investments.

The lowest LCOH is observed for designs with reference biomass. Here, the LCOH is 9–13% lower compared to design for reduced or no biomass, respectively. Furthermore, we observe a cost increase of up to 44% with increasing H_2_ demand. This cost increase is attributed to larger investments in electrolyzers, and CO_2_ capture, transport, and storage infrastructure, which are not installed in scenarios with lower H_2_ demands. Assuming that a pan-European CO_2_ transport infrastructure may be deployed independent from the decarbonization of the hard-to-abate industries investigated here, and therefore, CO_2_ transport infrastructure is available at little to no cost, H_2_ production capacities from SMR-CCS are expanded (Section S8). This is true, especially for scenarios with reference biomass availability Figure S6. Nevertheless, the impact of an inexpensive CO_2_ transport infrastructure is marginal with a LCOH cost reductions below 5% across scenarios.

If a different scenario materializes than the HSC was initially designed for, the HSC design has to be adjusted to the new conditions and the LCOH increases. Depending on the magnitude of those changes, it may not be possible to adapt the initial supply chain design such that the CO_2_ emission targets are fulfilled at all times (Section S6). HSC designs where the CO_2_ emission targets cannot be fulfilled at all times are considered infeasible. These infeasibilities occur predominantly when the H_2_ demand increases drastically (e.g., instead of min-low demands, high-max demands materialize) or when biomass availability is significantly overestimated during the design phase due to difficulties in scaling up the capacity of low-carbon H_2_ production and CO_2_ removal technologies.

The speed at which technology capacities can be expanded is described by the technology expansion rate as a function of the existing technology capacity (Section 2.4). The number of infeasible scenarios is lower for more conservative system designs, i.e., systems designed without biomass and for medium to max H_2_ demands, where larger shares of electrolyzers and CO_2_ removal technologies allow for a quicker scale-up of the already existing capacities. In contrast, supply chain designs that initially rely largely on biomass-based H_2_ production technologies are often unable to switch strategies and scale up the capacity of electrolyzers, SMR-CCS, and CO_2_ removal technologies quickly enough to meet the demand for low-carbon H_2_. Focusing initial investments and infrastructure development on biomass-based H_2_ production technologies can create a lock-in effect, delaying the scale-up of alternative H_2_ production technologies, even if they become more advantageous throughout the transition.

Higher technology expansion rates decrease the number of infeasible scenarios as they allow for a quicker expansion of the existing capacity and, thereby, offer greater flexibility to react and adapt the initial supply chain design to changes. Nevertheless, even for low and high technology expansion rates, changes in the investment strategies are small, and the minimum-regret strategy remains planning for medium H_2_ demand and without biomass. (Figure S2).

### 3.4 Minimum-Regret Solution

The minimum-regret solution is identified based on two criteria: (1) the solution results in the lowest LCOH in the worst scenario (min-max cost criteria), while (2) meeting the annual CO_2_ emissions targets (feasibility criteria). Overall, only three out of 15 design scenario designs meet the feasibility criteria of complying with the annual CO_2_ emissions target at all times and across all out-of-sample scenarios; these are the no biomass scenarios for medium to max H_2_ demands (Figure 6). Out of these three scenarios, the design for medium H_2_ demand and no biomass is identified as the minimum-regret solution as it results in the lowest LCOH in the worst case (Section 2.5). When relaxing the feasibility criteria and allowing for an overshoot of the annual CO_2_ emission targets as long as the net-zero emission target by 2050 is fulfilled, the number of potential minimum-regret designs increases to nine out of 15 designs (Figure S4). Nonetheless, designing HSCs for medium H_2_ demands without relying on biomass remains the minimum-regret solution, even when considering variations in the technology expansion rates (Figure S2).

In the minimum-regret scenario, about 63% of the H_2_ demand in 2050 is met via SMR-CCS, and about 34% via electrolysis from renewable electricity. Retrofitting SMR with CCS is a cost-effective approach to utilize existing production capacity while significantly reducing process emissions. It is assumed that natural gas will continue to be supplied via the existing infrastructure.

Depending on the H_2_ demand in the out-of-sample scenario, the electrolyzer capacity is expanded by up to 22 Mt/a. While planning for medium demands and reference biomass availability leads to similar LCOH across scenarios, achieving net-zero emissions by 2050 is increasingly challenging if H_2_ demands increase and reduced levels of biomass are available. Designing HSCs for medium H_2_ demands without relying on biomass is therefore identified as the minimum-regret strategy (Section 3.4).

A medium-sized H_2_ and CO_2_ transport infrastructure is built (3 Mt/a and 172 Mt/a, respectively, Figure 7). The benefit of investing in a medium-sized transport infrastructure is that it offers the flexibility to expand existing transport infrastructure and accommodate larger low-carbon H_2_ demands without oversizing transport infrastructure, thereby avoiding large stranded assets if significantly smaller low-carbon H_2_ demands materialize. Compared to other configurations, the minimum-regret transport infrastructure therefore maintains high network utilization rates (Figure S3).

**Figure 7:**
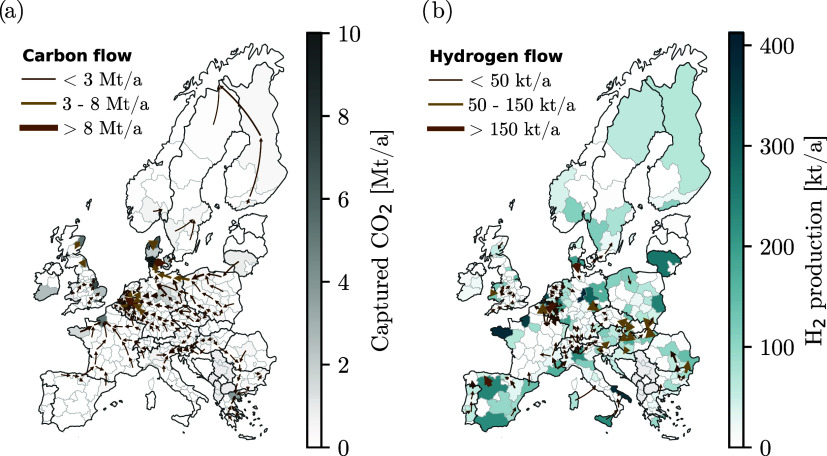
(a) H_2_ production and transport, and (b) CO_2_ capture and transport infrastructure in the minimum-regret scenario with no biomass and medium H_2_ demands. The minimum-regret strategy prioritizes the build-out of a medium-sized H_2_ and CO_2_ transport infrastructure.

Similar to ref 17, our findings suggest that connecting the regions of Belgium, The Netherlands, and the west of Germany with H_2_ and CO_2_ transport infrastructure is particularly valuable. For high H_2_ demands, it is cost-optimal to expand the transport infrastructure and invest in a European H_2_ and CO_2_ backbone infrastructure as refs 14,18,78 suggest. Higher low-carbon H_2_ demands may arise due to an increased need for low-carbon H_2_ in industry, or in other sectors, such as heavy transport and aviation.^67^

## 4 Discussion

### 4.1 The Role of Biomass in Decarbonizing Hydrogen Production

Biomass-based H_2_ production is identified as the most cost-effective low-carbon H_2_ production technology due to low H_2_ production cost and CO_2_ intensities. Especially in the initial phase of the transition, when the capital cost of electrolyzers is still high, biomass-based H_2_ production dominates the technology mix (Figure 4 and ref 51). This finding is consistent with ref 79, who investigate the cost-optimal H_2_ production technology mix under cost uncertainties. In comparison to biomass-based H_2_ production, the LCOH from electrolysis and renewable electricity from wind and solar electricity is 3–5 times higher, hindering the deployment of electrolyzers at large scales.^80^ Alternatively, electricity from nuclear or geothermal power could be used to produce low-carbon H_2_. Notwithstanding their lower electricity generation costs, cost estimates remain about 2 to 3 times higher than for biomass- or natural-gas-based alternatives.^26^

Even if the projected cost reductions of 2 €/kg_H_2__ can be achieved by 2040,^13^ the LCOH from electrolyzers is expected to remain high. Only SMR-CCS can achieve lower H_2_ production cost than biomass-based technologies (about 1.5 €/kg_H_2__), at the expense of higher process emissions (+2.5–3 kg_CO_2eq__/kg_H_2__). Therefore, the cost-competitiveness of electrolytic H_2_ is viewed as an unrealistic prospect in the medium term without appropriate policy support.^81^

However, biomass availability is uncertain as multiple sectors compete for it. For instance, biomass can be used as a fuel in the transport sector to decarbonize aviation or heavy-duty transport,^29,82^ or it can provide dispatchable, flexible energy in the power sector.^83^ While the LCOH is lower in scenarios that rely on biomass in their decarbonization strategy, planning without biomass leads to more flexible infrastructure designs.

Figure S4 visualizes the annual CO_2_ emissions for each design scenario. When planning without biomass, most scenarios can eventually achieve a net-zero supply chain design. Only when significantly higher H_2_ demands materialize than planned (see configurations for min-low demand), the annual CO_2_ emission targets are overshot during the transition. This observation holds across the investigated range of technology expansion rates (Figure S2).

If biomass is included in the technology mix, policymakers must ensure that biomass will be dedicated to low-carbon H_2_ production. Otherwise, it might not be possible to adapt the decarbonization strategy and scale up alternative H_2_ production and CO_2_ removal technologies quickly enough to satisfy the H_2_ demands in compliance with stricter CO_2_ emission limits (Figure 6).

Currently, biomass is predominantly used for heating and cooling (about 75% in 2018).^84^ However, existing studies indicate that using biomass to decarbonize transport and industry is more attractive than using biomass as a dispatchable, flexible energy source for electricity production.^70^ The current bioeconomy strategy of the EU focuses on increasing the sustainability and circularity, but does not provide clear guidelines on the use of biomass.^85^ Therefore, we recommend extending existing EU and national bioeconomy strategies to provide clear guidelines on the strategic use of the limited biomass resources; steering the use of biomass to sectors where biomass provides a cost-efficient decarbonization option and which lack alternatives.

### 4.2 The Role of Carbon Capture, Transport, and Storage

CO_2_ capture, transport, and storage (CCTS) infrastructure plays a central role in decarbonizing H_2_ production. More specifically, when coupling H_2_ production with CCS, large amounts of CO_2_ are captured at the production site, transported, and stored underground. When H_2_ production is based on electrolysis, CO_2_ removal technologies are installed to remove residual and upstream CO_2_ emissions. The CO_2_ removal technologies are typically located close to the CO_2_ storage sites to reduce transport costs, and thus, require less extensive CO_2_ transport infrastructure.

For scenarios with large H_2_ demands and reduced or no biomass, existing and planned CO_2_ storage sites are fully utilized to achieve net-zero H_2_ production. A reduced CO_2_ storage availability would lead to an increased installation of electrolyzers. Nevertheless, the model’s minimum cost solutions for minimum, medium, and maximum H_2_ demand indicate that about 7, 11, and 50 Mt_CO_2__/a CO_2_ removal and storage capacity are neededto offset upstream emissions and realize net-zero H_2_ production, respectively.

The need for CCTS infrastructure is widely recognized,^86^ and according to the International Association of Oil & Gas Producers (IOGP),^87^ Europe requires a minimum of 0.5–1 Gt_CO_2__/a of CO_2_ storage by 2050 to reach its net-zero emission targets. However, the capacity of existing and currently planned CO_2_ storage projects in Europe only corresponds to 13–26% of the storage capacity envisioned by the IOGP.^87^ Integrating CO_2_ utilization technologies into industrial value chains, e.g., to produce e-methanol or other e-fuels, could reduce the need for CO_2_ storage. However, given the limited scale and economic uncertainties surrounding this technology, it is unlikely to fully replace the need for dedicated CO_2_ storage.

Thus, while the currently announced CO_2_ storage capacity can support the decarbonization of industrial H_2_ demands, it may fall short of enabling decarbonization across multiple sectors. A coordinated strategy that combines storage expansion with investments in carbon utilization pathways could help address this gap and enhance the flexibility of future CO_2_ management strategies. Similar observations are made on a global scale, where only 10% of the required CO_2_ storage capacity might be available by 2050, considering the historically observed expansion rates for CO_2_ storage.^88^ This suggests that rapid changes and coordinated efforts are required to enable CCTS infrastructure across sectors and foster the energy transition.

### 4.3 Implications of a Constrained Technology Expansion

Technology expansion rates are computed based on historically observed annual growth rates, as they remain relatively stable over time.^49,71,89^ Previous studies suggest that energy systems models tend to overestimate the speed at which emerging technologies can penetrate the market.^49,90^ To address this, technology expansion constraints are added to limit the speed at which technology capacity can be expanded (Section 2.4).

The results indicate that the feasibility and cost of the out-of-sample designs strongly depends on the availability of CO_2_ removal technologies. The reduced investment in CO_2_ removal technologies in design scenarios that rely on biomass and the coupling with CCS poses challenges when biomass availability falls short and larger H_2_ demands materialize than initially planned. The results further indicate that it is important to plan ahead and invest in sufficient low-carbon H_2_ production capacity (about 10 Mt/a by 2030) to scale up low-carbon H_2_ production and facilitate larger low-carbon H_2_ demands. Otherwise, the transition might be delayed, and the decarbonization targets cannot be met (Figures 6 and S4).

Despite the EU’s high ambitions for H_2_, actions have been lacking, and it is unlikely that the 2030 green H_2_ targets will be met.^91^ By identifying low-regret investment opportunities, we aim to support decision-makers and help kick-start the development of low-carbon H_2_ supply chain infrastructure to enable the transition to net-zero emissions for hard-to-abate industry.

The planning horizon of 2022–2030 is especially critical. HSCs that plan for low or mean H_2_ demands often miss annual emission targets when larger H_2_ demands materialize due to insufficient low-carbon H_2_ production capacity, which delays the scale-up of SMR-CCS and electrolyzer capacity. Instead, larger shares of the existing, natural-gas-based SMR are deployed to meet the increased H_2_ demands.

Policies that introduce high carbon taxes or mandate specific low-carbon H_2_ production targets can stimulate demand and accelerate the adoption of low-carbon technologies.^92^ In addition, investor risks are reduced, encouraging investments. While these measures may not always be cost-optimal, system costs are expected to remain within 10% of the cost-optimum.^93^ The Renewable Energy Directive III (RED III)^10^ plays a key role in shaping the transition in Europe. By introducing binding renewable H_2_ generation targets for 2030 and 2035, the RED III can help create the renewable H_2_ demand needed to accelerate the adoption of electrolyzers. However, the effectiveness of these targets depends on complementary measures that increase the cost-competitiveness of electrolyzers in the short and medium term. Without additional policy support that incentivizes electrolyzer investments, the supply of renewable H_2_ is expected to remain scarce, increasing the risk of supply bottlenecks in the short term.^91^ Additionally, RED III focuses on H_2_ produced from renewable electricity. However, overlooking alternative low-carbon H_2_ production methods, such as SMR-CCS, can limit the flexibility in the transition. Retrofitting existing SMR capacity with CCS allows the continued use of the existing infrastructure while significantly reducing direct process emissions. The development of a more comprehensive European H_2_ strategy that includes concrete low-carbon H_2_ production targets but accounts for alternative low-carbon H_2_ production pathways could help mitigate these risks and ensure a cost-effective transition to net-zero emissions.

Lastly, the lack of a dedicated H_2_ and CO_2_ transport infrastructure,^23,94^ as well as uncertainties around material selection and process operation^86^ present additional barriers that delay infrastructure investments. While the creation of niche markets for low-carbon H_2_ could accelerate its adoption, this also necessitates a coordinated scale-up of the required low-carbon H_2_ and CCTS transport infrastructure.^23^

### 4.4 Central Modeling Assumptions

First, given the large uncertainty around future H_2_ demands, we focus on designing H_2_ transport infrastructure to decarbonize industrial H_2_ demands. However, in the future, additional low-carbon H_2_ demands may arise from shipping and other fuels or in the power sector. Moreover, we assume that the location of the industrial H_2_ demands does not change over time. This assumption is a simplification and neglects that investors may want to relocate their production to renewable-rich locations, such as the Iberian Peninsula, to save costs. Besides cost-benefits, political and societal factors impact investor decisions, making it challenging to account for demand relocation in the modeling.^95^

Second, we focus on modeling the transport of H_2_, CO_2_, biomass, and biomethane. We further assume that natural gas and grid electricity are available at each node. The existing natural gas and electricity grid infrastructure is not modeled. To produce H_2_ from renewable electricity, investments in dedicated wind and solar PV are needed to comply with the EU regulations for RFNBOs.^96^

Third, H_2_ and CO_2_ pipeline costs are far from linear, and economies of scale can significantly influence infrastructure project costs.^1^ However, only a small share of the system cost is attributed to H_2_ and CO_2_ transport (Figure 6), and changes due to variations in the transport costs are small (Figure S6). Previous investigations of the optimal infrastructure rollout for HSC found that investing in H_2_ and CO_2_ transport infrastructure remains cost-effective even when excluding pipelines due to their long construction times and only considering expensive H_2_ and CO_2_ trucks.^51^ Therefore, we expect our results to remain similar even when refining the representation of pipeline costs. Nonetheless, an in-depth assessment of these nonlinear cost effects on the transport infrastructure design could provide interesting insights and help refine transport infrastructure investment decisions.

Fourth, besides SMR-CCS, low-carbon H_2_ can be produced via autothermal reforming (ATR) coupled with CCS. While SMR represents the current industry standard in Europe,^12^ a recent paper by the Zero Emissions Platform^97^ suggests that ATR-CCS may be a promising alternative due to its higher CO_2_ capture rates. However, especially if larger H_2_ demands materialize, we observe a transition away from natural gas-based H_2_ production, which is in line with the EU’s ambition to reduce its dependence on natural gas.^4^ Therefore, the choice of reforming technology is not expected to alter the broader conclusions of this work.

Lastly, a yearly time resolution is used to maintain computational tractability. Following,^18,98^ we model industrial H_2_ demands and biomass availability as constant throughout the year. The capacity factors of wind and solar are approximated by their yearly average values. As a result, the seasonal and intradaily variability of wind and solar power is not captured, underestimating the production cost of electrolytic H_2_. Despite this, investment in electrolyzer capacity remains cost-disadvantageous in most scenarios. To allow for a detailed assessment, the temporal resolution of the optimization should be increased to represent the variable electricity supply from wind and solar and account for the need for energy storage.

### 4.5 Concluding Remarks

This work investigates the optimal infrastructure rollout of H_2_ supply chains (HSC) for European hard-to-abate industry that minimizes costs and achieves net-zero emissions. We consider a multiyear time horizon from 2022 to 2050 and include several H_2_ production pathways, namely water-electrolysis from renewable electricity, SMR from natural gas and biomethane, and biomass gasification. H_2_ production from biomass and natural gas can be coupled with CCS. In addition, CO_2_ removal technologies can be installed to remove residual CO_2_ emissions. Finally, CO_2_ and biomass transport infrastructure is designed alongside the HSC.

In addition, we account for uncertainties in the future H_2_ demand and the biomass availability. Following a a scenario-based approach, we define 15 scenarios, considering five levels of H_2_ demand and three levels of biomass availability. For each scenario, we determine the cost-optimal supply chain design­(design scenarios) and evaluate their performance in case alternative scenarios materialize (out-of-sample approach). Additional investments can be made to adapt the supply chain designs to the new operating conditions. However, these additional investments are limited by technology expansion constraints. The results are assessed based on the levelized cost of H_2_ and the ability to meet the annual CO_2_ emissions targets.

H_2_ production via SMR-CCS of natural gas and biomethane reforming are identified as the most cost-effective low-carbon H_2_ production pathways. Particularly attractive is the coupling of biomass-based H_2_ production with CCS, a process which achieves a CO_2_ removal, and can thereby offset emissions at other stages in the supply chain. Investments in electrolyzers are delayed due to high initial costs.

H_2_ transport infrastructure is built to decouple H_2_ production and demands. Furthermore, CO_2_ capture, transport, and storage infrastructure (CCTS) plays a central role in a low-carbon H_2_ economy. Especially for larger H_2_ demands, a well-developed European CCTS infrastructure is essential to mitigate emissions from H_2_ production and enable CCS. A minimum CO_2_ storage capacity of 2.7–50 Mt_CO_2__/a is needed in the model to decarbonize H_2_ production, emphasizing the need for coordinated efforts at the European level to accelerate the development of CCTS infrastructure.

Although incorporating biomass in the decarbonization strategy can reduce the LCOH between 9% to 13%, relying on biomass poses risks. If biomass supply falls short, scaling up alternative low-carbon H_2_ production technologies to adapt the supply chain design can be challenging, jeopardizing the decarbonization targets. Reducing the dependence on biomass-based H_2_ production mitigates this risk and enhances the system’s flexibility. By extending the EU bioenergy strategy and establishing clear guidelines on sectoral biomass allocation, policymakers can reduce uncertainty and guide investors. However, additional analyses are needed to identify the sectors where biomass provides the greatest value in the transition to net zero.

Conversely, dealing with the large uncertainty around the future low-carbon H_2_ demands is challenging. The results suggest that sufficient low-carbon H_2_ production capacity (about 10 Mt_H_2__/a by 2030) is needed to facilitate the scale-up of low-carbon H_2_ production capacity if larger H_2_ demands materialize. Delays in these early investments might result in a failure to achieve decarbonization targets, even if technology expansion rates are high.

By implementing concrete H_2_ production targets in the European H_2_ strategy, policymakers can help navigate these considerable uncertainties and provide clear directives on the envisioned role of low-carbon H_2_ in the transition. However, if the large uncertainty surrounding the future demand for low-carbon H_2_ pertains, this may further delay the scale-up of low-carbon HSCs.

Overlooking the importance of CCTS infrastructure can further delay the transition. The modeling results suggest that building H_2_ and CO_2_ transport infrastructure is cost-effective for achieving net-zero emission targets, regardless of the final H_2_ demand. Fostering investments in regional H_2_ and CO_2_ transport infrastructure that can be expanded if larger low-carbon H_2_ demands materialize can support a successful transition to net-zero H_2_ production in industry. Incentivizing investments in low-carbon H_2_ production capacities can further reduce the risks first-movers face (slow market dynamics, high low-carbon H_2_ production costs, lack of transport infrastructure) and facilitate the scale-up of low-carbon H_2_ markets.

## Supplementary Material



## Data Availability

The code and input data to reproduce the results presented in this work is available on Zenodo: 10.5281/zenodo.14288744.

## Notes

Input data to the optimization problem; assessment of the regional hydrogen demand uncertainty; mathematical formulation of the technology expansion constraints; sensitivity of the technology expansion constraint parameters; the network utilization rates, the annual carbon emissions; results of an optimiztic electrolysis scenario, results of an optimiztic carbon transport cost scenario, results of a scenario with a 90% decarbonization target (PDF)

## References

[1] IEA . The Future of Hydrogen, International Energy Agency, Tech. Rep., 2019. [Online]. Available https://iea.blob.core.windows.net/assets/9e3a3493-b9a6-4b7d-b499-7ca48e357561/The_Future_of_Hydrogen.pdf (visited on Aug 14, 2021).

[2] van
der Spek M., Banet C., Bauer C., Gabrielli P., Goldthorpe W., Mazzotti M., Munkejord S. T., Røkke N. A., Shah N., Sunny N., Sutter D., Trusler J. M., Gazzani M. (2022). Perspective on the hydrogen economy
as a pathway to reach net-zero CO 2 emissions in Europe. Energy Environ. Sci..

[3] O’Rourke P., Mignone B. K., Kyle P., Chapman B. R., Fuhrman J., Wolfram P., McJeon H. (2023). Supply and
Demand Drivers of Global
Hydrogen Deployment in the Transition toward a Decarbonized Energy
System. Environ. Sci. Technol..

[4] European Commission . REPowerEU: Joint European Action for more affordable, secure and sustainable energy EN. European Commission, Tech. Rep., 2022. [Online]. Available: 2022 https://eur-lex.europa.eu/legal-content/EN/TXT/?uri/COM%3A2022%3A230%3AFIN&qid/1653033742483. (visited on March 30, 2022).

[5] European Environment Agency . Annual European Union greenhouse gas inventory 1990–2021 and inventory report 2023. en, European Environment Agency, Brussels, Publication, Apr. 2023. [Online]. Available: https://www.eea.europa.eu/publications/annual-european-union-greenhouse-gas-2 (visited on Nov 16, 2023).

[6] EEA . Annual European Union greenhouse gas inventory 1990–2020 and inventory report 2022. Environmental Energy Agency, Technol. Rep., 2022, Issue: May. [Online]. Available: https://www.eea.europa.eu/publications/annual-european-union-greenhouse-gas-1.

[7] Paltsev S., Morris J., Kheshgi H., Herzog H. (2021). Hard-to-Abate Sectors:
The role of industrial carbon capture and storage (CCS) in emission
mitigation. Appl. Energy.

[8] Jones, N. Net-Zero Goals in Chemical Industry Could Shift Energy Demand, en-us, Mar. 2022. [Online]. Available: https://insight.factset.com/net-zero-goals-in-chemical-industry-could-shift-energy-demand (visited on Oct 6, 2023).

[9] European Commission . ’Fit for 55’: Delivering the EU’s 2030 Climate Target on the way to climate neutrality en, European Commission, Brussels, Tech. Rep., 2021. [Online]. Available: https://eur-lex.europa.eu/legal-content/EN/TXT/?uri/CELEX%3A52021DC0550 (visited on Sept 28, 2023).

[10] European Parliament and European Council . Directive - EU - 2023/2413 - EN - Renewable Energy Directive - EUR-Lex, en, Doc ID: 32023L2413 Doc Sector: 3 Doc Title: Directive (EU) 2023/2413 of the European Parliament and of the Council of 18 October 2023 amending Directive (EU) 2018/2001, Regulation (EU) 2018/1999 and Directive 98/70/EC as regards the promotion of energy from renewable sources, and repealing Council Directive (EU) 2015/652 Doc Type: L Usr_lan: en, 2023. [Online]. Available: https://eur-lex.europa.eu/eli/dir/2023/2413/oj/eng (visited on Feb 20, 2025).

[11] IEA . Global Hydrogen Review 2023, en, International Energy Agency, Technol. Rep., 2023. [Online]. Available: https://iea.blob.core.windows.net/assets/ecdfc3bb-d212-4a4c-9ff7-6ce5b1e19cef/GlobalHydrogenReview2023.pdf (visited on Jan 26, 2024).

[12] Fonseca, J. ; Muron, M. ; Pawelec, G. ; Yovchen, I. P. ; Kuhn, M. ; Fraile, D. ; Waciega, K. ; Azzimonti, M. ; Brodier, C. ; Alcade, I. ; Márton Ivan, A. ; Luca, L. ; Pafiti, A. ; Espitalier-Noel, M. ; Giusti, N. ; Durdevic, D. Clean Hydrogen Monitor 2023. Hydrogen Europe, Tech. Rep., 2023. [Online]. Available: https://hydrogeneurope.eu/wp-content/uploads/2023/10/Clean_Hydrogen_Monitor_11-2023_DIGITAL.pdf (visisted on Nov 22, 2023).

[13] Terlouw, W. ; Peters, D. ; van der Leun, K. Gas for Climate. The optimal role for gas in a net zero emissions energy system. Navigant, Technol. Rep., 2019. [Online]. Available: https://www.europeanbiogas.eu/wp-content/uploads/2019/11/GfC-study-The-optimal-role-for-gas-in-a-net-zero-emissions-energy-system.pdf (visited on June 30, 2022).

[14] Wang, A. ; David Mavins, J. J. ; Moultak, M. ; Leun, K. v. d. ; Peters, D. ; Buseman, M. Analysing future demand, supply, and transport of hydrogen. European Hydrogen Backbone, Technol. Rep., 2021, Issue: June. [Online]. Available: https://www.ehb.eu/files/downloads/EHB-Analysing-the-future-demand-supply-and-transport-of-hydrogen-June-2021-v3.pdf.

[15] Gabrielli P., Rosa L., Gazzani M., Meys R., Bardow A., Mazzotti M., Sansavini G. (2023). Net-zero emissions
chemical industry
in a world of limited resources. One Earth.

[16] European Commission . A hydrogen strategy for a climate-neutral Europe. European Commission, Technol. Rep., 2020, arXiv: 1011.1669v3 ISBN: 9788578110796 ISSN: 1098–6596, pp 1689–1699, [Online]. Available: https://ec.europa.eu/commission/presscorner/home/en.

[17] Agora Energiewende and AFRY Management Consulting . No-regret hydrogen: Charting early steps for H2 infrastructure in Europe. Agora Energiewende and AFRY Management Consulting, Technol. Rep., 2021. [Online]. Available: https://www.agora-energiewende.de/en/publications/no-regret-hydrogen/ (visited on March 12, 2022).

[18] Neumann, F. ; Zeyen, E. ; Victoria, M. ; Brown, T. The potential role of a hydrogen network in Europe, en, Joule, S2542435123002660, Jul. 2023, ISSN: 25424351. DOI: 10.1016/j.joule.2023.06.016. [Online]. Available: https://linkinghub.elsevier.com/retrieve/pii/S2542435123002660 (visited on July 21, 2023).

[19] Kotek P., Tóth B. T., Selei A. (2023). Designing a future-proof gas and
hydrogen infrastructure for Europe – A modelling-based approach. Energy Policy.

[20] Ochoa
Robles J., De-León Almaraz S., Azzaro-Pantel C. (2018). Design of
Experiments for Sensitivity Analysis of a Hydrogen Supply Chain Design
Model. Process Integr. Optim. Sustainability.

[21] Robles J. O., Azzaro-Pantel C., Aguilar-Lasserre A. (2020). Optimization of a hydrogen supply
chain network design under demand uncertainty by multi-objective genetic
algorithms. Comput. Chem. Eng..

[22] Zhen Z., Ou X., Wang Y., Zhou S. (2024). Assessing Transition Pathways of
Hydrogen Production in China with a Probabilistic Framework. Environ. Sci. Technol..

[23] Schlund D., Schulte S., Sprenger T. (2022). The who’s who of a hydrogen
market ramp-up: A stakeholder analysis for Germany. Renewable Sustainable Energy Rev..

[24] Gabrielli P., Charbonnier F., Guidolin A., Mazzotti M. (2020). Enabling low-carbon
hydrogen supply chains through use of biomass and carbon capture and
storage: A Swiss case study. Appl. Energy.

[25] Bauer C., Treyer K., Antonini C., Bergerson J., Gazzani M., Gencer E., Gibbins J., Mazzotti M., McCoy S. T., McKenna R., Pietzcker R., Ravikumar A. P., Romano M. C., Ueckerdt F., Vente J., van der Spek M. (2021). On the climate impacts of blue hydrogen production,. Sustainable Energy Fuels.

[26] Parkinson B., Balcombe P., Speirs J. F., Hawkes A. D., Hellgardt K. (2019). Levelized
cost of CO2 mitigation from hydrogen production routes. Energy Environ. Sci..

[27] Nevzorova T., Kutcherov V. (2019). Barriers to
the wider implementation of biogas as a
source of energy: A state-of-the-art review. Energy Strategy Rev..

[28] Schnorf V., Trutnevyte E., Bowman G., Burg V. (2021). Biomass transport for
energy: Cost, energy and CO2 performance of forest wood and manure
transport chains in Switzerland. J. Cleaner
Prod..

[29] Daioglou V., Wicke B., Faaij A. P. C., van Vuuren D. P. (2015). Competing
uses of biomass for energy and chemicals: Implications for longterm
global CO2 mitigation potential. GCB Bioenergy.

[30] Millinger M., Hedenus F., Zeyen E., Neumann F., Reichenberg L., Berndes G. (2025). Diversity of biomass usage pathways to achieve emissions
targets in the European energy system. Nat.
Energy.

[31] Pfenninger S., Hawkes A., Keirstead J. (2014). Energy systems
modeling for twenty-first
century energy challenges. Renewable Sustainable
Energy Rev..

[32] Blanco H., Leaver J., Dodds P. E., Dickinson R., García-Gusano D., Iribarren D., Lind A., Wang C., Danebergs J., Baumann M. (2022). A taxonomy of models for investigating hydrogen energy
systems. Renewable Sustainable Energy Rev..

[33] Yue X., Pye S., DeCarolis J., Li F. G., Rogan F., Gallachóir B. (2018). A review of
approaches to uncertainty assessment in energy system optimization
models. Energy Strategy Rev..

[34] Wen X., Jaxa-Rozen M., Trutnevyte E. (2023). Hindcasting to inform the development
of bottom-up electricity system models: The cases of endogenous demand
and technology learning. Appl. Energy.

[35] Koomey J., Craig P., Gadgil A., Lorenzetti D. (2003). Improving
Long-Range Energy Modeling: A Plea for Historical Retrospectives. Energy J..

[36] Craig P. P., Gadgil A., Koomey J. G. (2002). What Can
History Teach Us? A Retrospective
Examination of Long-Term Energy Forecasts for the United States*. Annu. Rev. Energy.

[37] Linstone H. A., Popper S. W., Bankes S. C. (2004). Shaping
the Next One Hundred Years:
New Methods for Quantitative, Long-Term Policy Analysis. Technol. Forecast. Soc. Change.

[38] Riera J. A., Lima R. M., Knio O. M. (2023). A review
of hydrogen production and
supply chain modeling and optimization. Int.
J. Hydrogen Energy.

[39] Li L., Wu N., Xie S., Meng C., Zheng Y., Wang X., Zhao Y. (2022). Review and
outlook on the international renewable energy development
- ScienceDirect. Energy Built Environ..

[40] Hugo A., Rutter P., Pistikopoulos S., Amorelli A., Zoia G. (2005). Hydrogen infrastructure
strategic planning using multi-objective optimization. Int. J. Hydrogen Energy.

[41] Almansoori A., Shah N. (2012). Design and operation
of a stochastic hydrogen supply chain network
under demand uncertainty. Int. J. Hydrogen Energy.

[42] Bique A. O., Maia L. K., Grossmann I. E., Zondervan E. (2022). Design of
hydrogen supply chains under demand uncertainty - a case study of
passenger transport in Germany. Process Syst.
Eng..

[43] Yang G., Jiang Y., You S. (2020). Planning and
operation of a hydrogen
supply chain network based on the off-grid wind-hydrogen coupling
system. Int. J. Hydrogen Energy.

[44] Fazli-Khalaf M., Naderi B., Mohammadi M., Pishvaee M. S. (2020). Design of a sustainable
and reliable hydrogen supply chain network under mixed uncertainties:
A case study. Int. J. Hydrogen Energy.

[45] Nunes P., Oliveira F., Hamacher S., Almansoori A. (2015). Design of
a hydrogen supply chain with uncertainty. Int.
J. Hydrogen Energy.

[46] Lou J., Liao Z., Jiang B., Wang J., Yang Y. (2014). Robust optimization
of hydrogen network. Int. J. Hydrogen Energy.

[47] De-León
Almaraz S., Azzaro-Pantel C., Montastruc L., Domenech S. (2014). Hydrogen supply chain optimization for deployment scenarios
in the Midi-Pyrénées region, France. Int. J. Hydrogen Energy.

[48] Aissi H., Bazgan C., Vanderpooten D. (2009). Min–max
and min–max
regret versions of combinatorial optimization problems: A survey. Eur. J. Oper. Res..

[49] Leibowicz B. D., Krey V., Grubler A. (2016). Representing
spatial technology diffusion
in an energy system optimization model. Technol.
Forecast. Soc. Change.

[50] eurostat . NUTS - Nomenclature of territorial units for statistics, 2021. [Online]. Available: https://ec.europa.eu/eurostat/web/nuts/overview (visited on April 7, 2024).

[51] Ganter A., Gabrielli P., Sansavini G. (2024). Near-term infrastructure rollout
and investment strategies for net-zero hydrogen supply chains. Renewable Sustainable Energy Rev..

[52] Ruiz, C. P. ; Nijs, W. ; Tarvydas, D. ; Sgobbi, A. ; Zucker, A. ; Pilli, R. ; Camia, A. ; Thiel, C. ; Hoyer-Klick, C. ; Dalla, L. F. ; Kober, T. ; Badger, J. ; Volker, P. ; Elbersen, B. ; Brosowski, A. ; Thrän, D. ; Jonsson, K. ENSPRESO - an open data, EU-28 wide, transparent and coherent database of wind, solar and biomass energy potentials, en, Jun. 2019. [Online]. Available: https://publications.jrc.ec.europa.eu/repository/handle/JRC116900 (visited on Nov 17,2023).

[53] Gonzalez-Aparicio, I. ; Zucker, A. ; Careri, F. ; Monforti, F. ; Huld, T. ; Badger, J. “EMHIRES dataset: Wind and solar power generation, en, Joint Research Center, Technol. Rep. May 2021. [Online]. Available: https://zenodo.org/record/4803353 (visited on Sept 28, 2023).

[54] IOGP . CCUS projects in Europe, IOGP, Technol. Rep., 2023. (visited on May 16, 2023).

[55] Ruiz Castello, P. ; Sgobbi, A. ; Wouter, N. ; Thiel, C. ; Francesco, Dalla. Longa. ; Kober, T. ; Berien Elbersen; Hengeveld, G. JRC-EU-TIMES model: bioenergy potentials for EU and neighbouring countries, Publications Office of the European Union, eng. LU, 2015, ISBN: 978–92–79–53879–7. [Online]. Available: https://data.europa.eu/doi/10.2790/39014 (visited on Nov 20, 2023).

[56] Scarlat N., Fahl F., Dallemand J. F., Monforti F., Motola V. (2018). A spatial
analysis of biogas potential from manure in Europe. Renewable Sustainable Energy Rev..

[57] Zorg Biogas GmbH . Biomethane transportation container 40” ISO Standard, May 2023. [Online]. Available: https://zorg-biogas.com/equipment/transportation-methane/methane-transportation-modules (visited on May 16, 2023).

[58] Material
Economics (2019). Industrial Transformation
2050 - Pathways to Net-Zero Emissions from EU Heavy Industry. Focus Catal..

[59] Luh S., Budinis S., Giarola S., Schmidt T. J., Hawkes A. (2020). Long-term
development of the industrial sector – Case study about electrification,
fuel switching, and CCS in the USA. Comput.
Chem. Eng..

[60] Azadnia A. H., McDaid C., Andwari A. M., Hosseini S. E. (2023). Green hydrogen supply
chain risk analysis: A european hard-to-abate sectors perspective. Renewable Sustainable Energy Rev..

[61] Krzywinski M., Altman N. (2014). Visualizing samples with box plots. Nat. Methods.

[62] Fils, G. ; Deutsch, M. 12 Insights on Hydrogen. Agora Energiewende and AFRY Management Consulting, Tech. Rep., 2021, p. 12 [Online]. Available: https://static.agora-energiewende.de/fileadmin/Projekte/2021/2021_11_H2_Insights/A-EW_245_H2_Insights_WEB.pdf.

[63] McWilliams, B. ; Zachmann, G. Navigating through hydrogen. Policy Cotnribution, Bruegel, 2021. [Online]. Available: https://www.bruegel.org/2021/04/navigating-through-hydrogen/.

[64] European Commission . Clean Planet for all: A European long-term strategic vision for a prosperous, modern, competitive and climate neutral economy, European Commission, Technol. Rep., 2018. [Online]. Available: https://eur-lex.europa.eu/legal-content/EN/TXT/?uri/CELEX%3A52018DC0773.

[65] Hebling, C. ; Ragwitz, M. ; Fleiter, T. ; Groos, U. ; Härle, D. ; Held, A. ; Jahn, M. ; Müller, N. ; Pfeifer, T. ; Plötz, P. ; Ranzmeyer, O. ; Schaadt, A. ; Sensfuß, F. ; Smolinka, T. ; Wietschel, M. EineWasserstoff-Roadmap für Deutschland, de, Fraunhofer-Institut für System- und Innovationsforschung ISI, Karlsruhe and Freiburg, Technol. Rep., 2019. [Online]. Available: https://www.ise.fraunhofer.de/content/dam/ise/de/documents/publications/studies/2019-10_Fraunhofer_Wasserstoff-Roadmap_fuer_Deutschland.pdf.

[66] Wachsmuth, J. ; Aydemir, A. ; Döscher, H. ; Eckstein, J. ; Proganietz, W. ; Francois, D. E. ; Scheer, D. The potential of hydrogen for decarbonising EU industry. European Parliamentary Research Service Scientific Foresight Unit (STOA) PE 697.199 – December 2021, Technol. Rep., 2021. 10.2861/271156. [Online]. Available: https://www.europarl.europa.eu/RegData/etudes/STUD/2021/697199/EPRS_STU(2021)69719_EN.pdf (visited on May 10, 2022).

[67] Fuel cells and hydrogen joint undertaking . Hydrogen Roadmap Europe: A Sustainable Pathway for the European Energy Transition. Fuel cells and hydrogen joint undertaking, Technol. Rep., 2019, Publication Title: Publications Office of the European Union. DOI: 10.2843/249013. [Online]. Available: https://op.europa.eu/en/publication-detail/-/publication/0817d60d-332f-11e9-8d04-01aa75ed71a1.

[68] Panoutsou, C. ; Maniatis, K. Sustainable biomass availability in the EU, to 2050 TAPPI J. 2021; Vol. 20 8 10.32964/tj20.8.

[69] Speirs J., McGlade C., Slade R. (2015). Uncertainty
in the availability of
natural resources: Fossil fuels, critical metals and biomass. Energy Policy.

[70] Wu F., Muller A., Pfenninger S. (2023). Strategic uses for ancillary bioenergy
in a carbon-neutral and fossil-free 2050 European energy system. Environ. Res. Lett..

[71] Mannhardt J., Gabrielli P., Sansavini G. (2024). Understanding the vicious cycle of
myopic foresight and constrained technology deployment in transforming
the European energy system. iScience.

[72] Mannhardt J., Gabrielli P., Sansavini G. (2023). Collaborative
and selfish mitigation
strategies to tackle energy scarcity: The case of the European gas
crisis. iScience.

[73] Gurobi Optimization LLC . Gurobi Optimizer Reference Manual. 2022. [Online]. Available: https://www.gurobi.com.

[74] European Commission . European Climate Law. 2021. [Online]. Available: https://eur-lex.europa.eu/legal-content/EN/TXT/?uri/CELEX:32021R1119.

[75] Ozkan M., Nayak S. P., Ruiz A. D., Jiang W. (2022). Current status and
pillars of direct air capture technologies. iScience.

[76] climeworks . Suppport the scale-up of direct air capture, en, 2023. [Online]. Available: https://climeworks.com/direct-air-capture?utm_source/googleBrand&utm_medium/cpc&utm_campaign/GS-AO-World-en-Brand&utm_term/climeworks&gclid/CjwKCAjwnOipBhBQEiwACyGLugmiA-HDfg1ekwyHv1jZ0nRSwOX7_qYgTdny4vRaR9cosBve55ElJxoCxAQQAvD_BwE (visited on Oct 23, 2023).

[77] Statistics Norway . Land use and land cover, en, Jul. 2023. [Online]. Available: https://www.ssb.no/en/natur-og-miljo/areal/statistikk/arealbruk-og-arealressurser (visited on Oct 25, 2023).

[78] Kountouris I., Bramstoft R., Madsen T., Gea-Bermúdez J., Münster M., Keles D. (2024). A unified European hydrogen infrastructure
planning to support the rapid scale-up of hydrogen production. Nat. Commun..

[79] Lane B., Reed J., Shaffer B., Samuelsen S. (2021). PEM Fuel cell
and electrolysis cell technologies and hydrogen infrastructure development
– a review. Energy Environ. Sci..

[80] Wang Y., Pang Y., Xu H., Martinez A., Chen K. S. (2022). PEM Fuel
cell and electrolysis cell technologies and hydrogen infrastructure
development – a review. Energy Environ.
Sci..

[81] Staffell I., Scamman D., Velazquez Abad A., Balcombe P., Dodds P. E., Ekins P., Shah N., Ward K. R. (2019). Role of carbon capture,
storage, and utilization to enable a Net-Zero-CO2-emissions aviation
sector. Ind. Eng. Chem. Res..

[82] Becattini V., Gabrielli P., Mazzotti M. (2021). Role of carbon capture, storage,
and utilization to enable a Net-Zero-CO2-emissions aviation sector. Ind. Eng. Chem. Res..

[83] Bogdanov D., Farfan J., Sadovskaia K., Aghahosseini A., Child M., Gulagi A., Oyewo A. S., de Souza
Noel Simas Barbosa L., Breyer C. (2019). Radical transformation pathway towards
sustainable electricity via evolutionary steps. Nat. Commun..

[84] European Commission . EU rules on sustainable biomass, en, 2024. [Online]. Available: https://energy.ec.europa.eu/topics/renewable-energy/bioenergy/biomass_en (visited on Jan 24, 2024).

[85] European Commission . Bioeconomy: the European way to use our natural resources: action plan 2018. Publications Office of the European Union, eng. LU, 2018, ISBN: 978–92–79–97443–4. [Online]. Available: https://data.europa.eu/doi/10.2777/79401 (visited on Sept 11, 2023).

[86] Bui M., Adjiman C. S., Bardow A., Anthony E. J., Boston A., Brown S., Fennell P. S., Fuss S., Galindo A., Hackett L. A., Hallett J. P., Herzog H. J., Jackson G., Kemper J., Krevor S., Maitland G. C., Matuszewski M., Metcalfe I. S., Petit C., Puxty G., Reimer J., Reiner D. M., Rubin E. S., Scott S. A., Shah N., Smit B., Trusler J. P. M., Webley P., Wilcox J., Dowell N. M. (2018). Carbon capture and storage (CCS): The way forward. Energy Environ. Sci..

[87] International Association of Oil & Gas Producers . Press Release, 2022. [Online]. Available: https://iogpeurope.org/news/europe-needs-a-co2-storage-ambition-for-2050/#::~text/Ahead%20of%20the%202nd,an%20interim%20ambition%20for%202035. (visited on June 12, 2023).

[88] Martin-Roberts E., Scott V., Flude S., Johnson G., Haszeldine R. S., Gilfillan S. (2021). Carbon capture and storage at the end of a lost decade. One Earth.

[89] Wilson, C. Meta-analysis of unit and industry level scaling dynamics in energy technologies and climate change mitigation scenarios. International Institute for Applied Systems Analysis (IIASA), Tech. Rep., 2009, pp. 1–7. [Online]. Available https://www.jstor.org/stable/resrep15764.3. (visited on Sept 10, 2023).

[90] Bertram C., Johnson N., Luderer G., Riahi K., Isaac M., Eom J. (2015). Carbon lock-in through capital stock inertia associated with weak
near-term climate policies. Technol. Forecast.
Soc. Change.

[91] Odenweller A., Ueckerdt F., Nemet G. F., Jensterle M., Luderer G. (2022). Probabilistic feasibility space of
scaling up green
hydrogen supply. Nat. Energy.

[92] Shi Y., Wei Z., Shahbaz M., Zeng Y. (2021). Exploring the dynamics of low-carbon
technology diffusion among enterprises: An evolutionary game model
on a two-level heterogeneous social network. Energy Econ..

[93] Greevenbroek, Kv. ; Schmidt, J. ; Zeyringer, M. ; Horsch, A. Little to lose: The case for a robust European green hydrogen strategy, arXiv.2412.07464 [eess], Dec. 2024, DOI: 10.48550/arXiv.2412.07464. [Online]. Available: http://arxiv.org/abs/2412.07464 (visited on March 17, 2025).

[94] IEA . Technology Perspectives Energy Special Report on Carbon Capture Utilisation and Storage CCUS in clean energy transitions, International Energy Agency, Tech. Rep., 2020. (visited on March 7, 2023).

[95] Verpoort P.
C., Gast L., Hofmann A., Ueckerdt F. (2024). Impact of global heterogeneity
of renewable energy supply on heavy industrial production and green
value chains. Nat. Energy.

[96] European Commission . Delegated regulation on Union methodology for RFNBO. Oct. 2023. [Online]. Available: https://energy.ec.europa.eu/system/files/2023-02/C_2023_1087_1_EN_ACT_part1_v8.pdf (visited on Oct 4, 2023).

[97] ZEP . CCS/CCU projects - Zero Emissions Platform, 2023. [Online]. Available: https://zeroemissionsplatform.eu/aboutccs-ccu/css-ccu-projects/ (visited on May 16, 2023).

[98] Victoria M., Zeyen E., Brown T. (2022). Speed of technological transformations
required in Europe to achieve different climate goals. Joule.

