# Supplementary material for: Minimum-Regret Hydrogen and Carbon Supply Chains to Decarbonize European Industrial Hydrogen Demands
Source: Environ Sci Technol. 2025 Jul 11;59(28):14372–87. doi: 10.1021/acs.est.4c13659 (PMC12288081; doi:10.1021/acs.est.4c13659)
Supplement: Supplementary file 1 [file es4c13659_si_001.pdf]

Supporting Information for

Minimum-Regret Hydrogen and Carbon Supply Chains to Decarbonize European Industrial Hydrogen Demands

Alissa Ganter[a], Paolo Gabrielli[a], Hanne Goericke[a,b], Giovanni Sansavini[a]

[a]*Institute of Energy and Process Engineering, Wilhelm-Johnen Straße, Zürich, 8092, Switzerland*
[b]*RWTH Aachen University, Templergraben 55, Aachen, 52062, North Rhine-Westphalia, Germany*

Summary: 15 pages, 4 tables, 7 figures.

S1. Input data to the optimization problem

The following details the input data assumptions of the hydrogen supply chain (HSC) model. S1.1 reports the energy carrier prices and carbon intensities. S1.2 provides an overview of the conversion and transport technology assumptions. Lastly, S1.3 summarizes the conditioning technology assumptions.

S1.1. Energy carrier prices and carbon intensities

The country-specific grid electricity prices and CO_2 intensities are taken from [1]. The country-specific natural gas prices are taken from [2]. The yearly evolution of the natural gas prices is estimated following [3]. The regional wet and dry biomass prices are taken from [4]. Finally, the CO_2 intensity of natural gas, wet biomass, and dry biomass are taken from [5], [6].

S1.2. Conversion and transport technologies

The parameters describing the technology cost of the conversion technologies are described in Tables S1 and S2. The parameters describing the performance of the conversion technologies are reported in Tables S3 and S4. The capacity factors and capacity limits for renewable energy technologies are taken from [7]. Finally, the techno-economic parameters of the transport technologies are described in Table S4. The transport distances for the transport technologies are approximated by the Haversine distance between the centroids of two neighboring regions.

Table S1: Capital investment costs for the available conversion technologies. bm = biomethane; e = electricity.

**Technologies**	unit	2022	2025	2030	2035	2040	2045	2050
Electrolysis [8], [9]	€/kW_{H_2}	1079	913	747	622	498	456	415
SMR [8]	€/kW_{H_2}	840	824	809	793	777	761	745
SMR-CCS [10]	€/kW_{H_2}	1551	1404	1256	1237	1219	1200	1182
Gasification [11], [12]	€/kW_{H_2}	1327	1287	1247	1207	1167	1128	1088
Gasification-CCS [11], [12]	€/kW_{H_2}	2449	2216	1983	1953	1924	1895	1866
Anaerobic digestion [9], [12]	€/kW_{bm}	1224	1164	1103	1074	1048	1019	993
Wind onshore [13]	€/kW_e	1353	1303	1252	1217	1182	1174	1165
Wind offshore [13]	€/kW_e	2115	2009	1903	1827	1751	1732	1712
Solar-PV (rooftop) [13]	€/kW_e	1364	1156	949	875	800	726	652
Solar-PV (utility-scale) [13]	€/kW_e	640	547	455	426	398	381	364
CO_2 removal [14]	€/(kg_{CO_2}/h)	7139	5225	3311	2816	2321	2133	1945
CO_2 storage [15]	€/(kg_{CO_2}/h)	210	210	210	210	210	210	210

Table S2: Fixed and variable operation and maintenance (0&M) costs, technology lifetime, and construction time (build time) for the available conversion technologies. bm = biomethane; e = electricity.

**Technologies**	**fix O&M** [%]	**var O&M** [€/kW_{H_2}]	**Lifetime** [years]	**Build time** [years]
Electrolysis [8]	1.5	0	10	2
SMR [8]	4.7	0	25	2
SMR-CCS [10]	3	0	25	2
Gasification [11], [12]	5	0	20	2
Gasification-CCS [11], [12]	5	0	20	2
Anaerobic digestion [9], [12]	6	0 €/kW_{bm}	20	2
Wind onshore [13]	1	14€/kW_e	30	2
Wind offshore [13]	2	39€/kW_e	30	2
Solar-PV (rooftop) [13]	2	12€/kW_e	40	2
Solar-PV (utility-scale) [13]	2	8 €/kW_e	40	2
CO_2 removal [14]	4	0 €/t_{CO_2}	25	2
CO_2 storage [15]	6	0 €/t_{CO_2}	40	2

Table S3: Technological parameters of the available production technologies. If the electricity balance is negative, electricity has to be provided to the system. g = natural gas or biomethane; e = electricity; B = dry biomass; b = wet biomass; bm = biomethane; LC = liquid CO_2.

**Production technologies**	**Conversion**	**Efficiency**	**CO_2 capture**	**CO_2 intensity**
Electrolysis [10]	e→H_2	0.64	-	-
SMR [5]	g → H_2<br>H_2 → e	0.77<br>0.041	-	-
SMR-CCS [5]	g → H_2<br>H_2 → e	0.77<br>0.016	90 %	-
Gasification [6]	B → H_2<br>H_2 → e	0.62<br>-0.093	-	-
Gasification-CCS [6]	B → H_2<br>H_2 → e	0.62<br>-0.153	57 %	-
Anaerobic digestion [6]	b → bm	0.435 t/MWh	-	-
CO_2 capture (retrofit) [5]	H_2 → H_2<br>e → H2	1<br>0.025	90 %	-
CO_2 storage [16]	LC → LC<br>e → LC	1<br>38 kWh/t	-	-1

Table S4: Techno-economic parameters of the available transport technologies. GH_2 = gaseous H_2; LH_2 = liquid H_2; LC = liquid CO_2, B = dry biomass; l = transport distance.

**Transport technologies**	**Carrier**	**Investment**	**O&M**	**Operation**	**Lifetime**	**Build time**	**CO_2 intensity**
		[€/kW km]	[%]	[€/(kW km)]	[year]	[year]	[g/(kWh km)]
H_2 truck (gas) [10]	GH_2	35 €/kWh	4	$5{\cdot}10^{-5}$	12	1	$5{\cdot}10^{-2}$
H_2 truck (liquid) [10]	LH_2	8 €/kWh	4	$8 \cdot 10^{-6}$	12	1	$8{\cdot}10^{-3}$
H_2 pipeline [10]	GH_2	3	4	0	40	4	0
Dry biomass truck [17]	B	6 €/kWh	4	$3{\cdot}10^{-4}$	12	1	$8{\cdot}10^{-3}$
CO_2 truck (liquid) [16]	LC	550 €/(t km)	4	0.5 €/(t km)	12	1	$7{\cdot}10^{-5}$ 1/km
CO_2 pipeline [16]	LC	3000 €/(t/h km)	1	0 €/(t km)	45	4	$1.6{\cdot}10^{-6}$ 1/km

S1.3. Hydrogen and carbon conditioning technologies

Due to its low energy density at ambient temperatures and pressures (2.4 kg/m^3 at 25°C and 30 bar), H_2 is typically transported as a compressed gas at ambient temperatures and high pressures (25°C, 200-350 bar), or as a liquid (-253°C, 1 bar)[18]–[20], requiring the installation of conditioning technologies in the form of H_2 compression, liquefaction, and evaporation technologies. Similar considerations apply to CO_2, which is therefore typically transported in its liquid form [16]. The conditioning technologies are modeled following [18] and [20]. The techno-economic parameters of the H_2 conditioning technologies are reported in [21].

S2. Regional hydrogen demand uncertainty

The regional H_2 demands for ammonia production, methanol production and refineries are estimated based on a regionally resolved dataset obtained from [22]. The regional H_2 demands for steel and cement industry are estimated using the annual emissions dataset published by the European Environmental Agency (EEA). The dataset includes the location and CO_2 emissions of European hard-to-abate industrial facilities emitting more than 0.1 Mt of CO_2 per year [23]. The emissions data for 2018 is used since the datasets for later years are incomplete. Based on the emissions data for 2018, we can derive the industry-specific CO_2 emissions per NUTS2 region in Europe. Assuming that industry-specific emissions are proportional to the production capacity, we can then estimate the regional H_2 demand.

We analyze the variability in the regional H_2 demand estimates by comparing their standard deviation. Fig. S1 visualizes the standard deviation for each region and industry in 2050, namely (a) ammonia production, (b) methanol production, (c) refineries, (d) cement production, and (e) steel production. The standard deviation is highest for steel and methanol production, reaching up to 250 kt/a in selected regions in the north-east of Germany and the north-west of France, and up to 280 kt/a in the west of the Netherlands, respectively. The standard deviation for ammonia production is substantially lower, exceeding 100 kt/a only in few select regions in the Netherlands, Germany, Poland and Lithuania. Cement and refineries exhibit the lowest standard deviations. The H_2 demands from refineries are expected to decline in future years due to reduced fossil fuel demands. Cement facilities are distributed across the NUTS2 regions and similar in size, resulting in standard deviations around 20 kt/a in most regions.

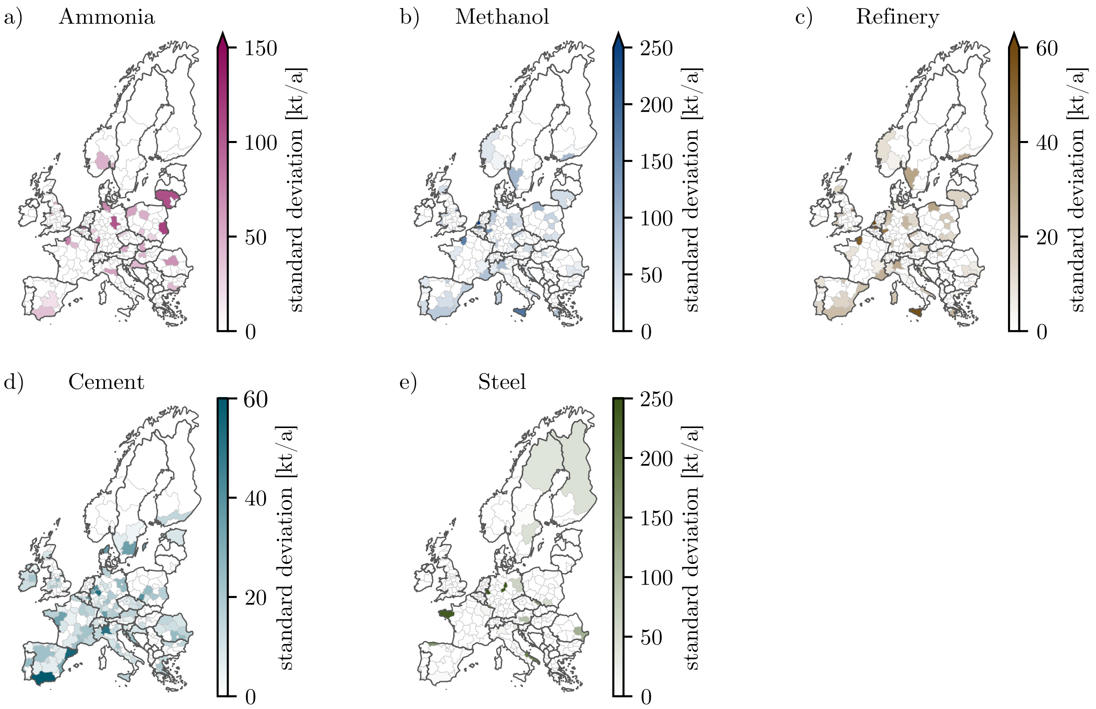

Figure S1: Regional standard deviation of the H_2 demand estimates for a) ammonia production, b) methanol production, c) refinery, d) cement production, and e) steel production.

S3. Technology expansion constraints

The capacity of technology $h \in \mathcal{H}$ at position $p \in \mathcal{P}$ and time t is expressed by $S_{h,p,t}$. Conversion technologies are installed at nodes $p = n \in \mathcal{N}$ and transport technologies can be installed at edges $p = e \in \mathcal{E}$. In each year, the technology capacity can be expanded by $\Delta S_{h,p,t}$. Existing technology capacities that are within their lifetime l_h are expressed by the parameter $s^{\text{ex}}_{h,p,\bar{t}}$:

$$S_{h,p,t} = \underbrace{\sum_{\tilde{t}=\max(0,t-l_h+1)}^{t} \Delta S_{h,p,\tilde{t}}}_{\text{Capacity increase}} + \underbrace{\sum_{\bar{t}=min(t-l_h+1,0)}^{0} s^{\text{ex}}_{h,p,\bar{t}}}_{\text{Capacity of existing technologies}} \ , \tag{C.1}$$

Furthermore, the technology capacity $S_{h,p,t}$ and the capacity expansion $\Delta S_{h,p,t}$ are constrained by $s^{\max}_h$, and $\Delta s^{\max}_h$, respectively:

$$0 \leq S_{h,p,t} \leq s^{\max}_h \tag{C.2}$$

$$0 \leq \Delta S_{h,p,t} \leq \Delta s^{\max}_h \tag{C.3}$$

In the out-of-sample approach, the technology capacity expansion is additionally constrained by the technology expansion constraints. These additional constraints are modeled and parameterized following [24], [25]. The technology expansion is limited by the technology expansion rate ϑ_h multiplied by the existing knowledge $K_{h,p,t}$, which

represents the expertise and knowledge of the industry (Eq. (C.7)). In addition, spillover effects from one country m to another $\tilde{\mathcal{M}} = \mathcal{M} \setminus \{m\}$ are considered, assuming a knowledge spillover rate $\omega = 0.07$. The unbounded market share ξ and the unbounded capacity addition ζ_h allow entry into niche markets [24]:

$$0 \leq \Delta S_{h,n,t} \leq (1 + \vartheta_h) \left(K_{h,m,t} + \omega \sum_{\tilde{m} \in \tilde{\mathcal{M}}} K_{h,\tilde{m},t} \right) + \xi \sum_{\tilde{h} \in \tilde{\mathcal{I}}} S_{\tilde{h},n,t} + \zeta_h, \tag{C.4}$$

Spillover effects are not included for transport technologies $h \in \mathcal{J} \subset \mathcal{H}$, which connect regions across all nodes:

$$0 \leq \Delta S_{h,e,y} \leq \vartheta_h K_{h,e,t} + \xi \sum_{\tilde{h} \in \tilde{\mathcal{J}}} S_{\tilde{h},e,t} + \zeta_h. \tag{C.5}$$

To avoid unrealistically high spillover effects, the cumulative capacity additions are constrained by the cumulative existing knowledge:

$$\sum_{p \in \mathcal{P}} \Delta S_{h,p,y} \leq \sum_{p \in \mathcal{P}} \left(\vartheta_h K_{h,p,t} + \xi \sum_{\tilde{h} \in \tilde{\mathcal{H}}} S_{\tilde{h},p,y} + \zeta_h \right). \tag{C.6}$$

where the existing knowledge $K_{h,p,t}$ is approximated by the previous capacity additions $\Delta S_{h,p,y}$ and $\Delta s^{\text{ex}}_{h,p,y}$, and depreciated over time with the knowledge depreciation rate $\delta = 0.1$:

$$K_{h,p,y} = \sum_{\tilde{t}=t_0}^{t-1} (1-\delta)^{(t-\tilde{t})} \Delta S_{h,p,\tilde{t}} + \sum_{\hat{t}=-\infty}^{\psi(t_0)} (1-\delta)^{\left(t+(\psi(t_0)-\hat{t})\right)} s^{\text{ex}}_{h,p,\bar{t}}. \tag{C.7}$$

S4. Sensitivity of the technology expansion constraint parameters

The technology expansion constraint limits the maximum annual growth rate of a technology, and is determined by the technology expansion rate and the existing capacity of a technology. Here, we investigate the impact of different expansion rates on the levelized cost of H_2 and compare the results for low expansion rates of 10 %, and high expansion rates of 29 % to the reference case of 20 %. Fig. S2 visualize the change in the levelized cost of H_2 (LCOH) for low and high expansion rates with respect to the reference case (Figure 6). A reduction in the technology expansion rate increases the systems inertia making it more difficult to quickly adapt the investment strategy and system cost increase (up to 1.5 €/kg). In contrast, an increase in the technology expansion rate reduces the system inertia, allowing for a quicker implementation of changes in the investment strategy, and a reduction in cost (up to 1.1 €/kg). In general, smaller systems are more sensitive to changes in the expansion rate (e.g., systems designed for min or low H_2 demand), whereas the impact of the expansion rate reduces for larger systems, where the maximum annual growth rates remains high due to the larger existing capacities in the system.

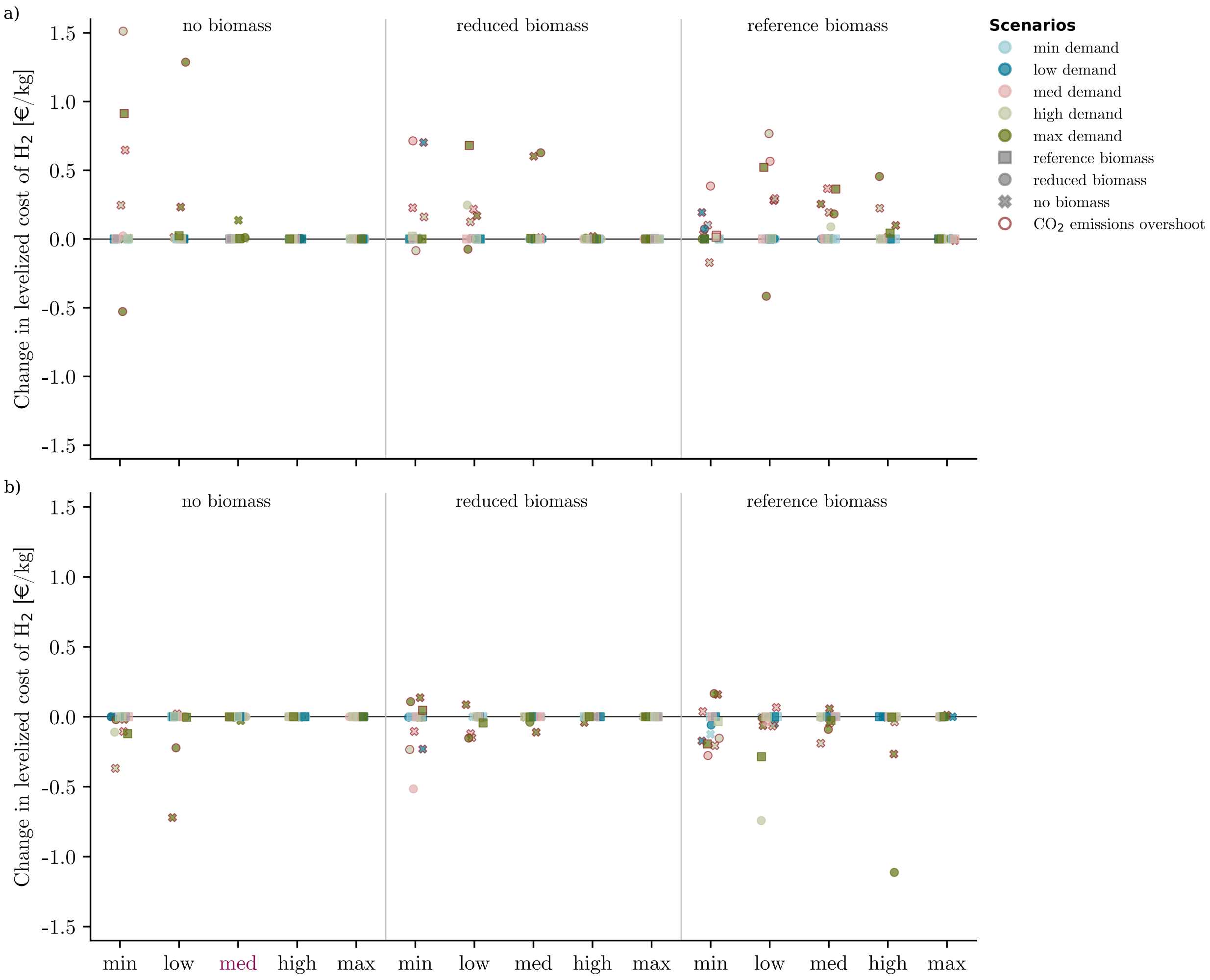

Figure S2: Change in levelized cost of H_2 (LCOH) for a) low (10 %) and b) high (29 %) technology expansion rates across all scenarios $s \in \mathcal{S}$ with respect to the reference case (20 %). The LCOH is computed as the net present cost divided by the net present H_2 production. The markers indicate the change in the LCOH with respect to the reference case presented in Fig. 6 that arises if the supply chain is initially designed for design scenario $a \in \mathcal{S}$, but out-of-sample scenario $b \in \mathcal{S}$ materializes. A red marker edge color indicates scenarios that are considered infeasible, as the CO_2 emissions target cannot be fulfilled at all times. The minimum-regret design is highlighted in purple.

S5. Network utilization

Fig. S3 shows the utilization rate of the H_2 and CO_2 transport networks in 2050 across the 15 design scenarios. The network utilization rate in 2050 is computed as the carrier flow divided by the available network capacity, and the variability within each design scenario stems from the 14 out-of-sample scenarios. In general, the mean network utilization rates are lower for higher H_2 demands. Supply chains that are initially designed for low H_2 demands are characterized by small, local transport networks, which are fully utilized across the out-of-sample scenarios. However, small H_2 and CO_2 transport capacities prohibit a quick expansion of the transport infrastructure. Instead, alternative H_2 production technologies have to be deployed, resulting in substantially higher supply chain costs or a failure to achieve the CO_2 emissions targets (Fig. S4).

In contrast, while building large, pan-European H_2 and CO_2 transport networks offers more flexibility, capacities often remain unused if lower H_2 demands materialize, and utilization rates plummet.

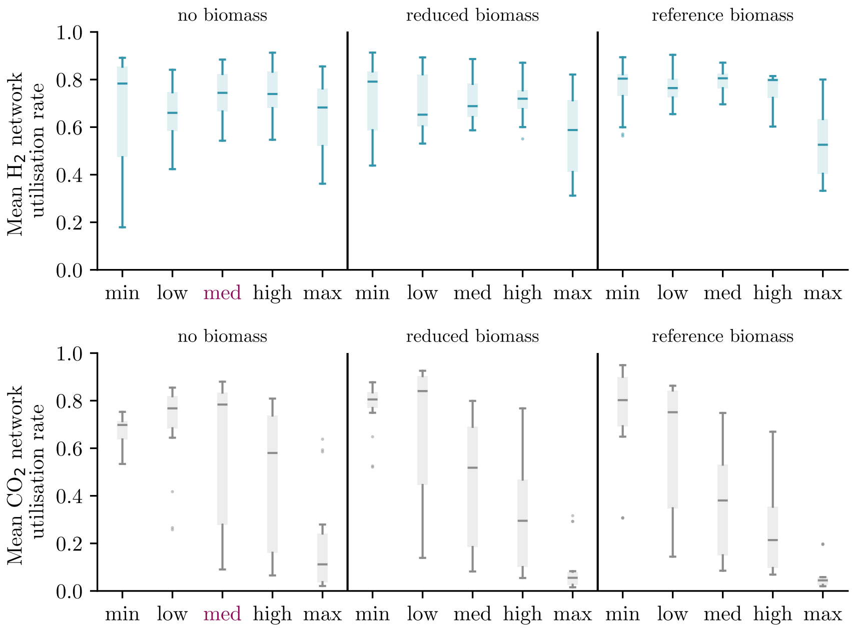

Figure S3: Boxplots of the mean network utilization rate in 2050 for the H_2 and CO_2 transport infrastructures for each design scenario. The minimum-regret design is highlighted in purple.

S6. Annual carbon emissions

Fig. S4 shows the mean annual CO_2 emissions in each design scenario. The grey area visualizes the area in which the annual CO_2 emissions are lower or equal to the annual CO_2 emissions target. In particular, supply chain designs for min and low H_2 demands often exceed the annual CO_2 emission limits, and low-carbon H_2 production has to be substituted with carbonaceous H_2 production to satisfy the H_2 demand. In contrast, supply chains designed

to accommodate larger H_2 demands are typically able to adapt their infrastructure quickly enough to achieve the climate targets. Here, we observe that, on average, the HSC emissions stay below the imposed annual CO_2 emission targets.

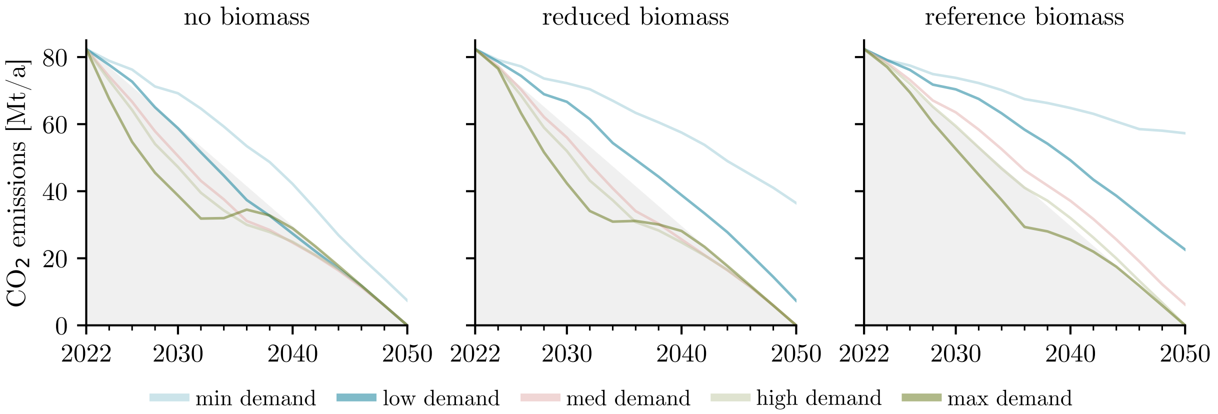

Figure S4: Mean annual CO_2 emissions for each design scenario design.

S7. Optimistic electrolysis scenario

We include an optimistic case for electrolyzers to investigate the impact of our input parameter assumptions on the investment decisions. To this end, the capital investment cost of the electrolyzers is reduced from 1079 €/kW_{H_2} in 2022 and 413 €/kW_{H_2} in 2050 to 985 €/kW_{H_2} in 2022 and 298 €/kW_{H_2} in 2050. In addition, the electrolysis lifetime is increased from 10 to 20 years. Fig. S5 visualizes the changes in the cost-optimal H_2 production capacities between 2022 and 2050 compared to the reference case presented in Fig. 4. Even in this optimistic case, the share of electrolyzers does not increase significantly with respect to the reference case, and changes remain below 8 % for the H_2 production capacities.

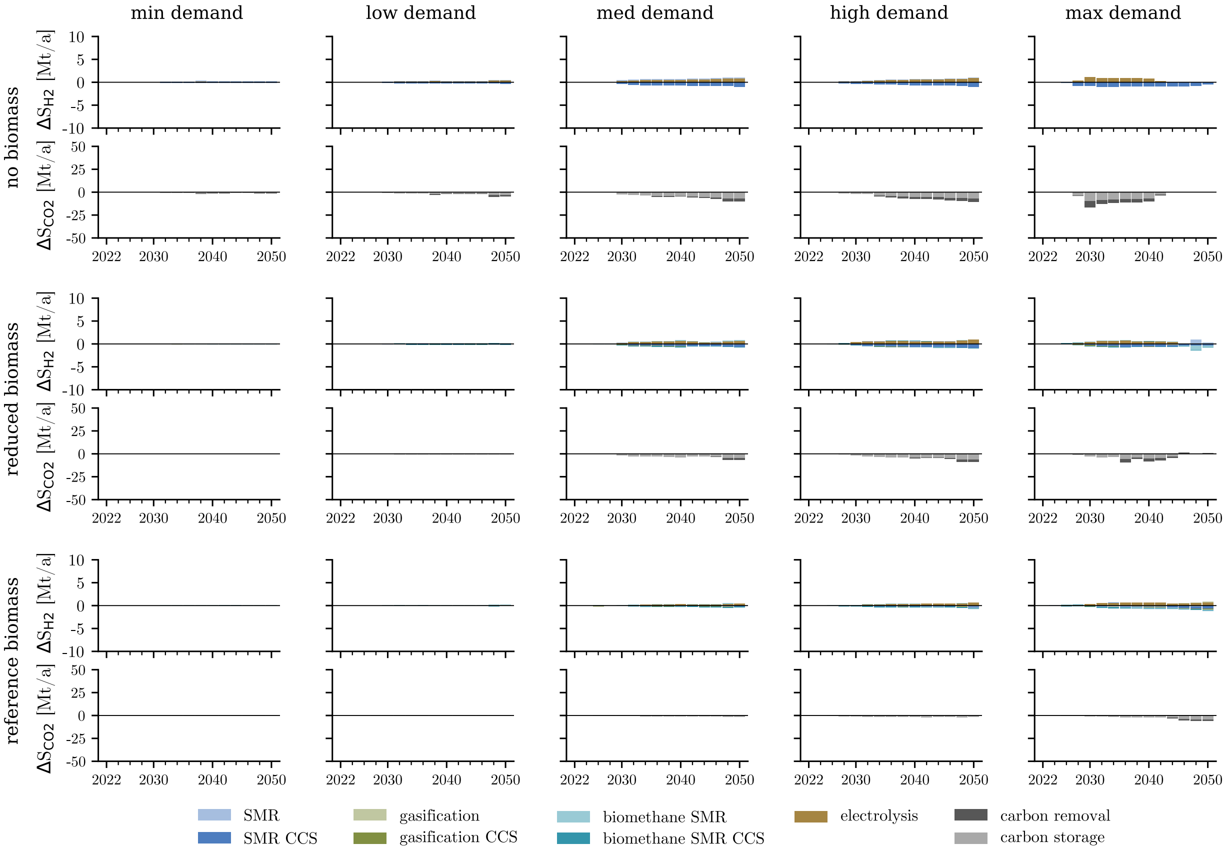

Figure S5: Changes in the cost-optimal H_2 production and CO_2 capture and storage capacities in Mt/a from 2022 to 2050 across all design scenarios for optimistic techno-economic electrolysis assumptions compared to the reference case presented in Fig. 4. H_2 production technologies include steam methane reforming (SMR) from natural gas, biomethane reforming, biomass gasification, and water-electrolysis from electricity. SMR, biomethane reforming, and biomass gasification can be coupled with CCS. In addition, CO_2 removal and CO_2 storage technologies can be installed.

S8. Optimistic carbon transport cost scenario

We include an optimistic case for CO_2 network to investigate the impact of our input parameter assumptions on investment decisions. To this end, the capital investment cost of the CO_2 trucks and pipelines is reduced to zero, and only the operational costs for CO_2 trucks (0.5 €/(t km)) remain. Fig. S6 visualizes the changes in the cost-optimal H_2 production capacities between 2022 and 2050 with respect to the reference case presented in Fig. 4. In design scenarios with biomass availability, we observe a shift from low-carbon H_2 production from biomass-based H_2 production to H_2 production from natural gas coupled with CCS. Design scenario designs with med-max H_2 demands are affected the most, where biomass-based H_2 production capacities reduce by up to 16 % while SMR-CCS capacities increase by up to 26 %. Furthermore, investments in CO_2 removal and CO_2 storage capacities occur earlier in time, and CO_2 storage capacities increase up to 15 % (see reference biomass, max demand). Scenarios that do not include biomass are less affected, and changes remain below 10 % and 15 % for H_2 production capacities and CO_2 capture and storage capacities, respectively.

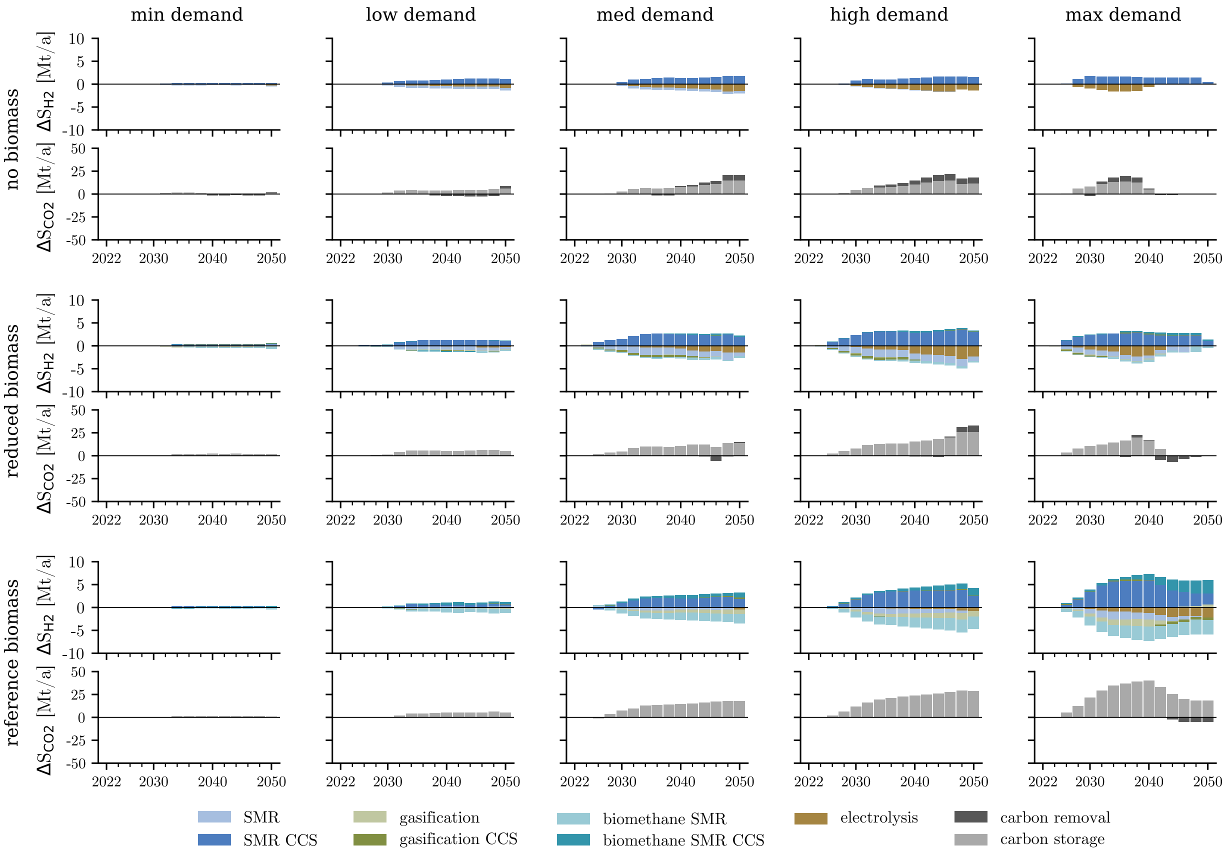

Figure S6: Changes in the cost-optimal H_2 production and CO_2 capture and storage capacities in Mt/a from 2022 to 2050 across all design scenarios for optimistic CO_2 transport cost assumptions compared to the reference case. H_2 production technologies include steam methane reforming (SMR) from natural gas, biomethane reforming, biomass gasification, and water-electrolysis from electricity. SMR, biomethane reforming, and biomass gasification can be coupled with CCS. In addition, CO_2 removal and CO_2 storage technologies can be installed.

S9. 90 % decarbonization scenario

We include a case with a reduced decarbonization target of 90 % by 2050 to investigate the impact of the net-zero emissions target on our investment decisions. Fig. S7 visualizes the changes in the cost-optimal H_2 production capacities between 2022 and 2050 compared to the reference case presented in Fig. 4. We observe that even for a reduced decarbonization target of 90 % by 2050, CO_2 capture and storage infrastructure is required. However, on average, CO_2 capture and storage capacities reduce by about 24 %.

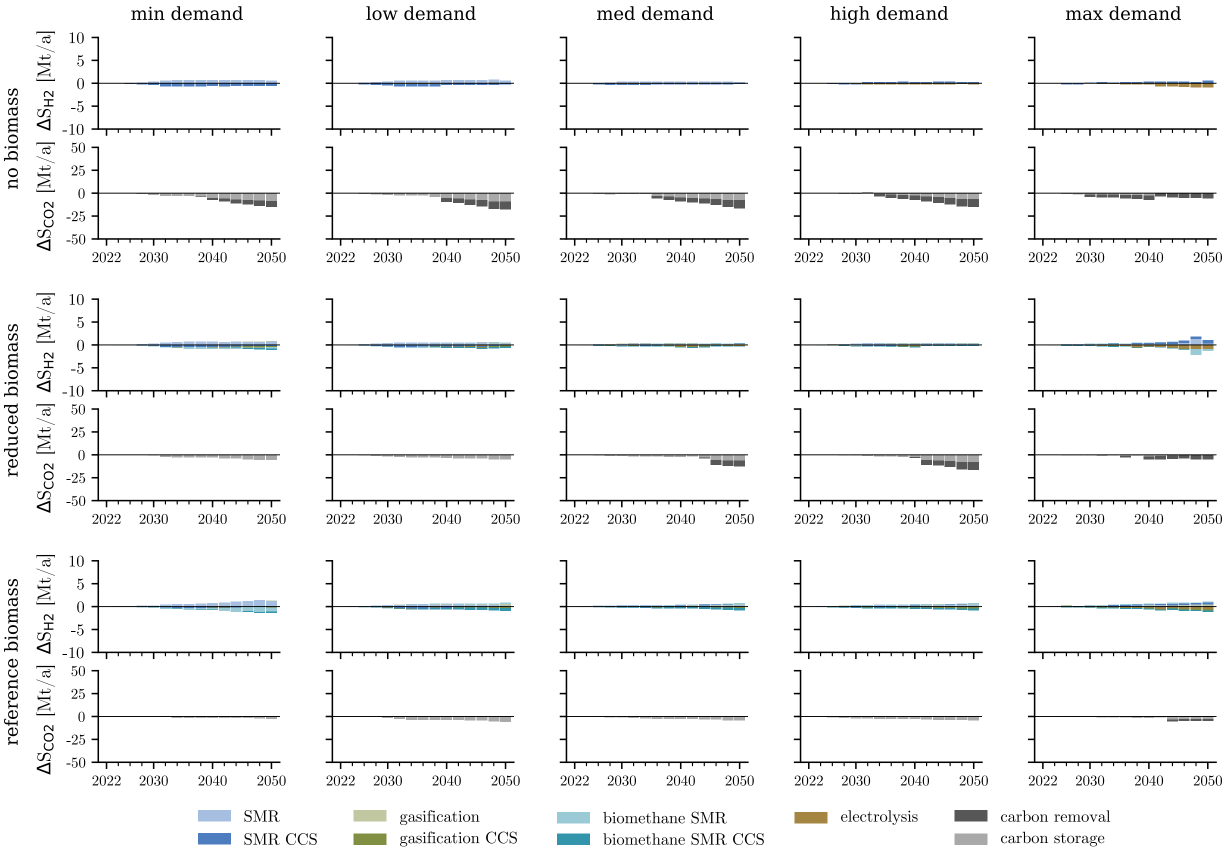

Figure S7: Changes in the cost-optimal H_2 production and CO_2 capture and storage capacities in Mt/a from 2022 to 2050 across all design scenarios for a 90 % decarbonization target by 2050 compared to the reference case reported in Fig. 4. H_2 production technologies include steam methane reforming (SMR) from natural gas, biomethane reforming, biomass gasification, and water-electrolysis from electricity. SMR, biomethane reforming, and biomass gasification can be coupled with CCS. In addition, CO_2 removal and CO_2 storage technologies can be installed.

References

[1] P. Capros, A. De Vita, A. Florou, M. Kannavou, T. Fotiou, P. Siskos, I. Tsiropoulos, N. Katoufa, I. Mitsios, S. Evangelopoulou, G. Asimakopoulou, T. Kalokyris, L. Paroussos, K. Fragkiadakis, P. Karkatsoulis, L. Höglund-Isaksson, W. Winiwarter, P. Purohit, A. Gómez-Sanabria, P. Rafaj, L. Warnecke, A. Deppermann, M. Gusti, S. Frank, P. Lauri, F. di Fulvio, N. Forsell, Havlík Petr; P. Witzke, and M. Kesting, "EU Reference Scenario 2020," European Commission, Tech. Rep., 2021, ISBN: 9789276393566, p. 184. [Online]. Available: https://op.europa.eu/s/shWr.

[2] eurostat, *Gas prices components for non-household consumers - annual data*, 2022. [Online]. Available: https://ec.europa.eu/eurostat/databrowser/view/NRG_PC_203_C$DEFAULTVIEW/default/table (visited on 12/06/2022).

[3] IEA, "Renewable Energy Market Update - Outlook for 2023 and 2024," en, International Energy Agency, Tech. Rep., 2023. [Online]. Available: https://iea.blob.core.windows.net/assets/63c14514-6833-4cd8-ac53-f9918c2e4cd9/RenewableEnergyMarketUpdate_June2023.pdf (visited on 09/28/2023).

[4] C. P. Ruiz, W. Nijs, D. Tarvydas, A. Sgobbi, A. Zucker, R. Pilli, A. Camia, C. Thiel, C. Hoyer-Klick, L. F. Dalla, T. Kober, J. Badger, P. Volker, B. Elbersen, A. Brosowski, D. Thrän, and K. Jonsson, *ENSPRESO - an open data, EU-28 wide, transparent and coherent database of wind, solar and biomass energy potentials*, en, Jun. 2019. [Online]. Available: https://publications.jrc.ec.europa.eu/repository/handle/JRC116900 (visited on 11/17/2023).

[5] C. Antonini, K. Treyer, A. Streb, M. van der Spek, C. Bauer, and M. Mazzotti, "Hydrogen production from natural gas and biomethane with carbon capture and storage – A techno-environmental analysis," *Sustainable Energy & Fuels*, vol. 4, no. 6, pp. 2967–2986, 2020, ISSN: 2398-4902. DOI: 10.1039/D0SE00222D. [Online]. Available: http://xlink.rsc.org/?DOI=D0SE00222D.

[6] C. Antonini, K. Treyer, E. Moioli, C. Bauer, T. J. Schildhauer, and M. Mazzotti, "Hydrogen from wood gasification with CCS – a techno-environmental analysis of production and use as transport fuel," *Sustainable Energy & Fuels*, vol. 5, no. 10, pp. 2602–2621, 2021, ISSN: 2398-4902. DOI: 10.1039/D0SE01637C. [Online]. Available: http://xlink.rsc.org/?DOI=D0SE01637C.

[7] I. Gonzalez-Aparicio, A. Zucker, F. Careri, F. Monforti, T. Huld, and J. Badger, "EMHIRES dataset: Wind and solar power generation," en, Joint Research Center, Tech. Rep., May 2021. [Online]. Available: https://zenodo.org/record/4803353 (visited on 09/28/2023).

[8] IEA, "The Future of Hydrogen," International Energy Agency, Tech. Rep., 2019. [Online]. Available: https://iea.blob.core.windows.net/assets/9e3a3493-b9a6-4b7d-b499-7ca48e357561/The_Future_of_Hydrogen.pdf (visited on 08/14/2021).

[9] M. Victoria, E. Zeyen, and T. Brown, "Speed of technological transformations required in Europe to achieve different climate goals," *Joule*, vol. 6, no. 5, pp. 1066–1086, 2022, arXiv: 2109.09563 Publisher: Elsevier Inc., ISSN: 25424351. DOI: 10.1016/j.joule.2022.04.016. [Online]. Available: https://doi.org/10.1016/j.joule.2022.04.016.

[10] IEA, "IEA G20 Hydrogen report: Assumption," International Energy Agency, Tech. Rep., 2020. [Online]. Available: https://iea.blob.core.windows.net/assets/29b027e5-fefc-47df-aed0-456b1bb38844/IEA-The-Future-of-Hydrogen-Assumptions-Annex_CORR.pdf (visited on 06/03/2022).

[11] M. Binder, Kraussler, Michael, Kuba, Matthias, and Luisser, Markus, "Hydrogen from biomass gasification," IEA Bioenergy, Tech. Rep., 2018, ISBN: 9781910154595. [Online]. Available: https://www.ieabioenergy.com/wp-content/uploads/2019/01/Wasserstoffstudie_IEA-final.pdf (visited on 07/01/2022).

[12] W. Terlouw, D. Peters, and K. van der Leun, "Gas for Climate. The optimal role for gas in a net zero emissions energy system," Navigant, Tech. Rep., 2019. [Online]. Available: https://www.europeanbiogas.eu/wp-content/uploads/2019/11/GfC-study-The-optimal-role-for-gas-in-a-net-zero-emissions-energy-system.pdf (visited on 06/30/2022).

[13] A. Kättlitz, M. C. Cavarretta, N. Buyuk, O. Lebois, and P. Boersma, “Scenario Building Guidelines,” entsog and entso, Tech. Rep., 2021. (visited on 07/06/2022).

[14] M. Fasihi, O. Efimova, and C. Breyer, “Techno-economic assessment of CO2 direct air capture plants,” *Journal of Cleaner Production*, vol. 224, pp. 957–980, Jul. 2019, ISSN: 09596526. DOI: 10.1016/j.jclepro.2019.03.086. [Online]. Available: https://linkinghub.elsevier.com/retrieve/pii/S0959652619307772.

[15] ZEP, “The Costs of CO2 Storage,” Zero Emissions Platform, Tech. Rep., 2011. [Online]. Available: www.zeroemissionsplatform.eu/library/publication/168-zep-cost-report-storage.html (visited on 05/06/2022).

[16] V. Becattini, P. Gabrielli, C. Antonini, J. Campos, A. Acquilino, G. Sansavini, and M. Mazzotti, “Carbon dioxide capture, transport and storage supply chains: Optimal economic and environmental performance of infrastructure rollout,” *International Journal of Greenhouse Gas Control*, vol. 117, p. 103 635, Jun. 2022, ISSN: 1750-5836. DOI: 10.1016/j.ijggc.2022.103635. [Online]. Available: https://www.sciencedirect.com/science/article/pii/S1750583622000548 (visited on 09/28/2023).

[17] V. Schnorf, E. Trutnevyte, G. Bowman, and V. Burg, “Biomass transport for energy: Cost, energy and CO2 performance of forest wood and manure transport chains in Switzerland,” *Journal of Cleaner Production*, vol. 293, p. 125 971, Apr. 2021, ISSN: 0959-6526. DOI: 10.1016/j.jclepro.2021.125971. [Online]. Available: https://www.sciencedirect.com/science/article/pii/S0959652621001918 (visited on 09/28/2023).

[18] P. Gabrielli, F. Charbonnier, A. Guidolin, and M. Mazzotti, “Enabling low-carbon hydrogen supply chains through use of biomass and carbon capture and storage: A Swiss case study,” *Applied Energy*, vol. 275, p. 115 245, Oct. 2020, ISSN: 0306-2619. DOI: 10.1016/j.apenergy.2020.115245. [Online]. Available: https://www.sciencedirect.com/science/article/pii/S0306261920307571 (visited on 08/23/2023).

[19] S. Krasae-in, J. H. Stang, and P. Neksa, “Development of large-scale hydrogen liquefaction processes from 1898 to 2009,” *International Journal of Hydrogen Energy*, vol. 35, no. 10, pp. 4524–4533, 2010, Publisher: Elsevier Ltd, ISSN: 03603199. DOI: 10.1016/j.ijhydene.2010.02.109. [Online]. Available: http://dx.doi.org/10.1016/j.ijhydene.2010.02.109.

[20] M. Reuß, T. Grube, M. Robinius, P. Preuster, P. Wasserscheid, and D. Stolten, “Seasonal storage and alternative carriers: A flexible hydrogen supply chain model,” *Applied Energy*, vol. 200, pp. 290–302, Aug. 2017, Publisher: Elsevier Ltd, ISSN: 03062619. DOI: 10.1016/j.apenergy.2017.05.050. [Online]. Available: https://linkinghub.elsevier.com/retrieve/pii/S0306261917305457 (visited on 04/24/2022).

[21] A. Ganter, P. Gabrielli, and G. Sansavini, “Near-term infrastructure rollout and investment strategies for net-zero hydrogen supply chains,” *Renewable and Sustainable Energy Reviews*, vol. 194, p. 114 314, Apr. 2024, ISSN: 1364-0321. DOI: 10.1016/j.rser.2024.114314. [Online]. Available: https://www.sciencedirect.com/science/article/pii/S1364032124000376 (visited on 04/04/2024).

[22] FCHO, “2022 hydrogen supply capacity and demand,” Fuel Cells and Hydrogen Observatory, Tech. Rep., Mar. 2022. [Online]. Available: https://observatory.clean-hydrogen.europa.eu/tools-reports/observatory-reports.

[23] EEA, “Annual European Union greenhouse gas inventory 1990–2020 and inventory report 2022,” Environmental Energy Agency, Tech. Rep., 2022, Issue: May. [Online]. Available: https://www.eea.europa.eu/publications/annual-european-union-greenhouse-gas-1.

[24] J. Mannhardt, P. Gabrielli, and G. Sansavini, “Understanding the vicious cycle of myopic foresight and constrained technology deployment in transforming the European energy system,” English, *iScience*, vol. 27, no. 12, Dec. 2024, Publisher: Elsevier, ISSN: 2589-0042. DOI: 10.1016/j.isci.2024.111369. [Online]. Available: https://www.cell.com/iscience/abstract/S2589-0042(24)02594-X (visited on 12/06/2024).

[25] B. D. Leibowicz, V. Krey, and A. Grubler, “Representing spatial technology diffusion in an energy system optimization model,” en, *Technological Forecasting and Social Change*, vol. 103, pp. 350–363, Feb. 2016, ISSN: 0040-1625. DOI: 10.1016/j.techfore.2015.06.001. [Online]. Available: https://www.sciencedirect.com/science/article/pii/S0040162515001675 (visited on 07/26/2023).
